# The role of alpha-lactalbumin in modulating tryptophan metabolism and serotonin synthesis

**DOI:** 10.1038/s41538-025-00497-6

**Published:** 2025-07-03

**Authors:** Shannon Shoff, Xuan He, Hanna Lee, Zhichao Zhang, Darya O. Mishchuk, Gulustan Ozturk, Daniela Barile, Merete Lindberg Hartvigsen, Anne Staudt Kvistgaard, Carolyn M. Slupsky

**Affiliations:** 1 Department of Nutrition, University of California-Davis, Davis, CA USA; 2 Department of Food Science and Technology, University of California-Davis, Davis, CA USA; 3 Arla Foods Ingredients Group P/S, Viby, Denmark; 4 Present Address: Department of Food Science & Nutrition, Cal Poly, San Luis Obispo, CA USA; 5 Present Address: Department of Food Science, University of Wisconsin-Madison, Madison, WI USA

**Keywords:** Metabolomics, Biochemistry

## Abstract

Tryptophan, critical for infant neurodevelopment, is limited in infant formulas. Tryptophan-rich α-lactalbumin is abundant in human milk but limited in bovine milk, and its metabolism in developing infants remains unclear. We developed an α-lactalbumin-enriched formula and conducted a feeding study with neonatal piglets to comprehensively monitor tryptophan utilization across serum, urine, liver, and brain using gas chromatography mass spectrometry (GC-MS) and nuclear magnetic resonance (NMR)-based metabolomics. Enrichment of α-lactalbumin led to higher circulating tryptophan and increased serotonin levels in the striatum. It also increased metabolic products from the kynurenine and indole pathways, which were predominantly excreted in urine. Despite these increases, the activity of the kynurenine pathway in the liver was lower, possibly mediated by reduced circulating cortisol, thus increasing brain tryptophan availability and favoring serotonin synthesis. These findings provide mechanistic insights that can guide the development of infant formulas to better mimic the metabolic profile of breastfed infants.

## Introduction

Human milk is the recommended source of nutrition for developing infants. However, when breastfeeding is not possible, infant formula is the most suitable alternative. Despite continuous advancements informed by human milk research, differences in protein concentration and composition between breast milk and formula still exist. Human milk contains 9–11 g/L of protein^1,2^, whereas formula, typically derived from cow’s milk, contains higher protein levels and differs in the total amino acid profile. This discrepancy is thought to contribute to the observed growth and metabolic differences between breastfed and formula-fed infants^3–7^.

The controversy over the optimal protein content in infant formula remains to be addressed. Although there is consensus that the protein content in typical formula exceeds the protein needs of infants, the safety threshold for reducing protein content, while ensuring adequate levels of essential amino acids like tryptophan, is not well-established. α-Lactalbumin constitutes ~22% of the total protein in human milk but is only 3.5% in bovine milk^8^. A typical infant formula uses bovine whey protein concentrate, which is low in α-lactalbumin but high in β-lactoglobulin, a protein not found in human milk^8^. Recent advances in dairy technology have led to the isolation of whey fractions with a high level of α-lactalbumin. Enriching infant formula with bovine α-lactalbumin, which has a similar amino acid composition to human α-lactalbumin, could partially bridge the differences between human milk and infant formula by increasing limiting amino acids, thus allowing for a reduction in overall protein content to more closely resemble the amino acid profile of human milk^9^.

In addition to its role as a source of essential amino acids, α-lactalbumin is believed to offer several potential health benefits, including antimicrobial properties, enhancement of immune function, and absorption of zinc and calcium^9^. Clinical trials have confirmed that formulas enriched with α-lactalbumin are safe, support adequate growth, increase energetic efficiency, and improve gastrointestinal tolerance in infants^10–18^.

Additionally, adequate dietary tryptophan is essential for infant cognitive and brain development, as it serves as a precursor for serotonin biosynthesis within the brain^19^. At the blood-brain barrier, tryptophan uptake competes with other large neutral amino acids (LNAAs), including branched-chain amino acids (BCAAs), phenylalanine, tyrosine, and methionine, via the large neutral amino acid transporter 1, which preferentially transports BCAAs and exhibits a lower affinity for tryptophan^20–22^. Typical formula contains higher levels of LNAAs, resulting in elevated circulating levels of these amino acids in formula-fed infants compared to breastfed infants^5,7,23,24^. Elevated levels of circulating BCAAs could potentially influence the uptake of tryptophan by the brain, leading to reduced neuronal serotonin synthesis and serotonergic signaling^25^. Thus, we hypothesized that an infant diet enriched with α-lactalbumin would increase the circulating tryptophan:LNAA ratio, potentially enhancing tryptophan influx into the brain and serotonin production.

Domestic pigs (*Sus domesticus*) have proven to be effective models for studying infant nutrition due to their similar digestive physiology, hormonal regulation, and neurodevelopmental trajectories^26,27^. The protein digestibility in 3-week-old piglets is comparable to that of 3-month-old human infants^28^, thus supporting the use of piglets as a translational model for understanding protein utilization in human infants.

In this study, our aim is to elucidate the underlying metabolic impacts of consumption of an α-lactalbumin-enriched formula at a molecular level and investigate how tryptophan from α-lactalbumin is utilized by hepatic tissue and four brain regions that are essential for learning, memory formation, language, reward processing, as well as appetite and sleep regulation. By investigating the developmental consequences of consuming formulas with varying whey protein compositions in neonatal piglets, we seek to generate mechanistic evidence that will inform the design of infant formulas to align closely with the growth and metabolic performance of breastfed infants.

## Results

### Characterization of sow milk and piglet formulas

Given the distinct nutritional requirements and differences in whey-to-casein ratios between pig milk and human milk, it is unsuitable to feed human infant formula directly to growing piglets. To develop an appropriate formula that aligns with the nutritional profile of sow milk, we analyzed pooled milk samples collected from a lactating sow between postpartum Days 6 and 14. We quantified total amino acids and measured major and trace minerals as well as other small-molecular-weight polar metabolites. Water-soluble nutrients such as lactose, choline, citrate, taurine, free amino acids, and *myo*-inositol were measured using ^1^H-NMR, with results summarized in Supplementary Table 1. Total amino acids were quantified following acid or alkaline hydrolysis using an amino acid analyzer equipped with ion-exchange chromatography, as shown in Supplementary Table 2. Minerals were measured using inductively coupled plasma mass spectrometry (ICP-MS) and are reported in Supplementary Table 3.

**Fig. 1:**
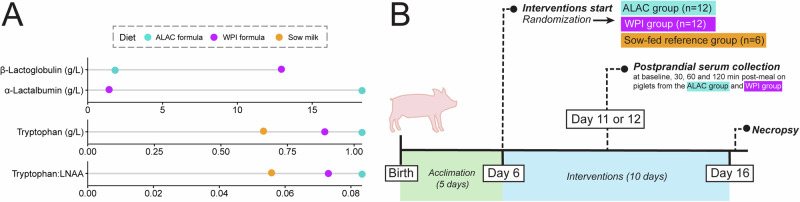
Formula feeding trial design, and the whey protein composition and tryptophan content of the diets. **A** Differences in α-lactalbumin, β-lactoglobulin, tryptophan content, and the tryptophan to other large neutral amino acid (LNAA) ratio among the α-lactalbumin-enriched formula (ALAC formula), the formula predominantly high in β-lactoglobulin (WPI formula), and sow milk. Detailed nutritional composition of the ALAC formula, the WPI formula, and sow milk is presented in Supplementary Tables 2–4. LNAA includes isoleucine, leucine, valine, phenylalanine, and tyrosine. **B** Overview of the 10-day formula-feeding study design. The study enrolled 30 piglets who suckled from their sows until postnatal Day 6. These piglets were then randomized into three groups: the ALAC group, which received the ALAC formula (*n* = 12); the WPI group, which received the WPI formula (*n* = 12); and a sow-fed reference group (*n* = 6). From Day 6 to 16, the ALAC and WPI groups were bottle-fed every 3 h, while the sow-fed group remained with the sow. On Days 11 and 12, postprandial blood measurements were collected from the ALAC and WPI piglets, with half of the piglets in each group being tested each day. On Day 16, the piglets were anesthetized and sacrificed ~1 h after their last meal. Blood, urine, liver, and brain samples were then collected for analysis. Of the total 30 piglets, one piglet was euthanized 2 days after the start of the feeding intervention due to an accidental injury caused by the sow.

Two isocaloric piglet formulas, designed to mimic the nutritional composition of sow milk, were developed and produced at the Milk Processing Laboratory at UC Davis. Each formula contained the same protein concentration but incorporated different sources of bovine whey protein that were predominantly high in either α-lactalbumin (ALAC formula) or β-lactoglobulin (WPI formula) (Supplementary Table 2). These formulas included lactose, protein, and lipids, as well as minerals, vitamins, free amino acids, taurine, and *myo*-inositol to prevent deficiencies (Supplementary Tables 2–4). To meet the essential amino acid requirements of growing piglets and ensure amino acid levels were comparable to those found in sow milk, the amino acid composition of each formula was carefully adjusted with a small amount of free amino acids (<8% of the total amino acids). Notably, free tryptophan was not added to these formulas; thus, tryptophan content was derived solely from the whey protein sources used. Following production, each batch of formula underwent microbiological testing for foodborne pathogens. A subsequent analysis of total amino acids and protein via SDS-PAGE confirmed that the ALAC formula was enriched in α-lactalbumin, resulting in a higher tryptophan content and tryptophan:LNAA ratio compared to the WPI formula (Fig. 1A, results detailed in Supplementary Table 2). These differences in tryptophan levels between the ALAC and WPI formulas were anticipated to significantly influence circulating tryptophan levels and affect tryptophan metabolism in the liver and brain.

### Growth of piglets fed the two formulas compared to the sow-fed reference group

To align with the practice of human formula feeding, 30 piglets stayed with their sows for the first 5 days post-birth to consume colostrum. We conducted a feeding trial from postnatal Day 6 to 16 (Fig. 1B). On Day 6, they were stratified by weight, sex, and litter and then randomly assigned to either continue with the sow’s milk (SF reference group) or to start one of two piglet formulas: the ALAC formula or the WPI formula (ALAC or WPI groups, respectively). To transition from sow’s milk to formula, all formula-fed piglets were comforted with a warm towel, cuddled, and hand-fed using nursing bottles at regular intervals. Additionally, to support normal social development and minimize separation stress, formula-fed piglets were group-housed according to the formula they received.

Despite being fed every 3 h with a volume of formula designed to meet their daily caloric needs, formula-fed piglets consistently showed slower growth compared to their SF counterparts (Supplementary Fig. 1A). This pattern of slower growth has been observed in piglets that are schedule-fed, as opposed to those with *ad libitum* access to piglet formula^29^. Additionally, this discrepancy may be attributed in part due to the formulas containing slightly lower calories and protein than sow milk (Supplementary Table 2). Initially, both ALAC and WPI groups showed a marked increase in formula intake over the first 2 days, adapting to bottle feeding and overcoming the initial stress of maternal separation. By Day 9, the intake of both groups met and exceeded the caloric requirements necessary for growth. On Day 11 or 12, postprandial blood draws were conducted, which led to a noticeable decrease in formula intake (Supplementary Fig. 1B). Although the volume of blood drawn was determined and approved by an experienced veterinarian based on body weight, the venipuncture itself may have contributed to changes in eating behavior subsequent to the procedure. Furthermore, this reduction could also be partly due to a 5-h fasting period prior to blood draws, causing the piglets to miss a meal. Despite compensatory feeding that was provided afterward, appetite was notably affected. Nevertheless, despite lower body weight gain compared to other SF counterparts of similar age and sex, none of these formula-fed piglets met the “failure to thrive” criteria, which is characterized by no weight gain for 2 weeks^29^.

While the formula-fed piglets experienced slower weight gain, their growth trajectories generally paralleled those of the SF piglets. Throughout the feeding period, they exhibited normal energy and activity levels, with veterinary personnel closely monitoring for any visual signs of infection to confirm their overall health. From Days 11 to 13, the ALAC group recovered their appetite faster than the WPI group (Supplementary Fig. 1B). To determine whether anemia could be present and affect the growth disparities, serum hemoglobin and hematocrit levels were assessed at the end of the study (Day 16). The results indicated no significant differences in hemoglobin and hematocrit levels among groups (Supplementary Fig. 2). This suggests that the observed differences in growth rates and appetite are unlikely to be caused by anemia.

To gain a preliminary understanding of whether diet may affect organ development, we evaluated the weights of the brain, liver, and kidneys on Day 16. Liver and kidney weights were significantly higher in SF piglets than in those receiving either the ALAC or WPI formulas. Variations in brain weight were also observed among the groups. However, with adjustment of body weight, both brain and kidney weights were significantly greater in the ALAC and WPI piglets compared to the SF piglets. The relative liver weight was notably higher in the ALAC piglets compared to the WPI piglets, although no significant differences were found between these groups and the SF piglets (Supplementary Table 5). Overall, body weight throughout the feeding intervention and organ weights at sacrifice showed no significant differences between piglets consuming either formula.

### Hormonal and metabolic responses following consumption of an α-lactalbumin-enriched formula

To investigate the postprandial metabolic response to an α-lactalbumin-enriched formula, blood samples were collected from piglets on Day 11 or 12 following over a 5-h fasting time, as well as at 30, 60, and 120 min after formula feeding. Due to the low availability of repeated measurement samples from all piglets at all timepoints, statistical analysis was evaluated cross-sectionally at each individual timepoint. Furthermore, additional samples were taken on Day 16 when piglets were euthanized at an average of 60 min post-feeding. To approximate the same postprandial timing as the formula-fed piglets, oxytocin was injected ~90 min prior to necropsy to stimulate milk letdown. Unfortunately, the precise timing and volume of sow milk intake are not known.

We first evaluated circulating ghrelin, a known marker of appetite, which is generally influenced by food intake. Following formula ingestion, we observed a significant decrease in circulating acylated and desacylated ghrelin levels between the baseline and 30 min post-meal samples, which indicates a normal physiological response of ghrelin in the postprandial state. However, there were no significant and consistent trends in both acylated and desacylated ghrelin levels between the two formula-fed groups at any time point, before or during the postprandial measurements, or at necropsy (Supplementary Fig. 3A, B).

Contrasting with acylated ghrelin, glucagon-like peptide-1 (GLP-1), which accelerates gastric emptying and facilitates rapid nutrient transit through the intestine^30^, showed a tendency toward lower levels in the ALAC group at baseline and 120 min post-feeding compared to the WPI group (Supplementary Fig. 3C). Furthermore, since GLP-1 stimulates insulin release from the pancreas in response to food intake^30^ and insulin is the key regulator of tryptophan and LNAA uptake in the brain^22^, serum insulin was assessed. The ALAC group showed a trend toward lower insulin at 60 min post-meal on Day 11 or 12 compared to the WPI group. However, insulin was not significantly different between the groups when evaluated again on Day 16 (Supplementary Fig. 3D).

### Postprandial tryptophan metabolic markers following α-lactalbumin-enriched formula

**Fig. 2:**
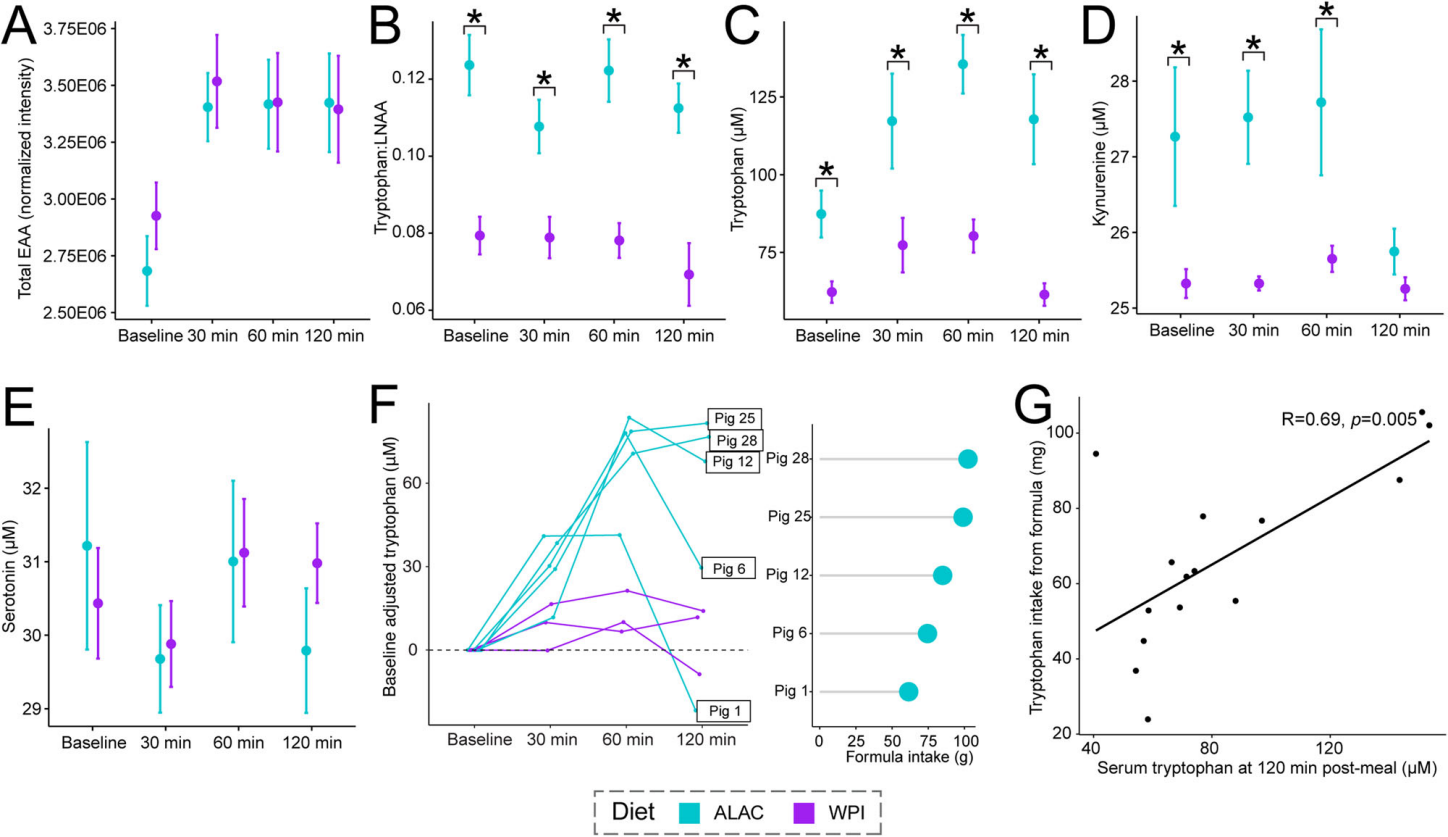
Postprandial response of formulas predominantly containing α-lactalbumin or β-lactoglobulin as major whey proteins affecting tryptophan metabolism, using blood samples drawn from the cephalic vein. **A**, **B** Levels of total essential amino acids (EAA) and the tryptophan to other large neutral amino acid (LNAA) ratio at baseline and at 30, 60, and 120 min post-meal. EAA measured include histidine, isoleucine, leucine, lysine, methionine, phenylalanine, threonine, tryptophan, and valine. LNAA includes isoleucine, leucine, valine, phenylalanine, and tyrosine. GC-MS-based untargeted metabolomics data were used. **C**–**E** Concentrations of tryptophan, kynurenine, and serotonin at baseline and at 30, 60, and 120 min post-meal. GC-MS-based targeted metabolomics data were used. **A**–**E** For the ALAC group, the number of samples analyzed at each time point was: baseline (*n* = 10), 30 min (*n* = 9), 60 min (*n* = 10), and 120 min (*n* = 6). For the WPI group: baseline (*n* = 10), 30 min (*n* = 8), 60 min (*n* = 10), and 120 min (*n* = 9). Data are represented as mean ± SEM. The significant differences between the ALAC and WPI groups were evaluated cross-sectionally using ANOVA on generalized log-transformed data, with litter effects accounted for in the model. *p < 0.05. **F** Postprandial tryptophan response by piglet using 4 repeated measures. Results were adjusted by baseline tryptophan level. Unadjusted results are presented in Supplementary Fig. 5. Formula intake for ALAC piglets is shown. **G** Pearson’s correlation demonstrates a strong positive correlation between tryptophan intake and serum tryptophan levels at 120 min post-meal.

To further understand how tryptophan from an α-lactalbumin-enriched formula was utilized, serum samples on Day 11 or 12 were first assessed via an untargeted metabolomics approach. Cluster-based analysis revealed differences in the overall serum metabolic profile at baseline and postprandial timepoints (Supplementary Fig. 4). As anticipated, the sum of all circulating essential amino acids increased postprandially, yet levels at 120 min did not return to the baseline level (Fig. 2A).

As the ALAC diet had higher tryptophan content and a higher tryptophan:LNAA ratio, ALAC piglets demonstrated higher serum tryptophan: LNAA ratios compared to piglets in the WPI group (Fig. 2B). Subsequent targeted quantification of tryptophan and tryptophan-derived metabolites (kynurenine and serotonin) revealed that ALAC piglets had elevated baseline serum tryptophan and maintained this higher level postprandially (Fig. 2C). Although baseline levels of serum kynurenine were significantly higher in the ALAC piglets, it did not exhibit a profound postprandial response when compared to tryptophan (Fig. 2D). Additionally, there were no significant differences in serum serotonin levels either at baseline or across postprandial timepoints (Fig. 2E). These results suggest that a tryptophan-enriched diet can significantly affect systemic tryptophan metabolism. Further exploration of this diet-driven impact will be detailed in the next section.

We performed a subsequent analysis on a subset of samples that had repeated measurements across all four timepoints. The result confirmed the finding observed in the cross-sectional evaluation (Fig. 2F, Supplementary Fig. 5). Furthermore, within the ALAC group, there was notable individual variation in postprandial tryptophan levels. Specifically, piglets that consumed more formula exhibited a delayed and prolonged elevation in postprandial tryptophan levels at 120 min post-meal, the final timepoint in our postprandial assessment, compared to those that consumed less formula. To explore this further, we assessed the relationship between the amount of tryptophan ingested from the formula and the circulating tryptophan levels at 120 min postprandially. A significant positive correlation was found (Fig. 2G), indicating that circulating tryptophan levels are directly influenced by the quantity of tryptophan in the diet in a time- and dose-dependent manner.

### Tryptophan from α-lactalbumin-enriched formula altered systemic tryptophan metabolism

To further understand the metabolic effects of consuming an α-lactalbumin-enriched formula, we performed an integrative analysis with measurements of tryptophan and tryptophan-derived metabolites using a combination of ^1^H-NMR and GC-MS-based targeted quantification in serum, urine, liver, and four brain regions important for learning, memory, appetite regulation and reward processing (hippocampus, hypothalamus, prefrontal cortex, and striatum). To account for variations in pre-necropsy formula intake and postprandial time, we used ANOVA to assess the differences between the two formula-fed groups, controlling for litter, formula intake at the meal prior to sacrifice, and time since meal in the serum and liver metabolite data. However, when comparing formula-fed piglets to SF piglets, control over feeding time and volume was not possible; thus, only the litter effect was controlled in these comparisons within the serum and liver datasets. In the datasets for urine and brain, the analysis controlled solely for the effect of litter. Overall, the serum, urine, liver, and brain metabolomes of SF piglets demonstrated distinct differences compared to those of the formula-fed piglets (Supplementary Fig. 6).

**Fig. 3:**
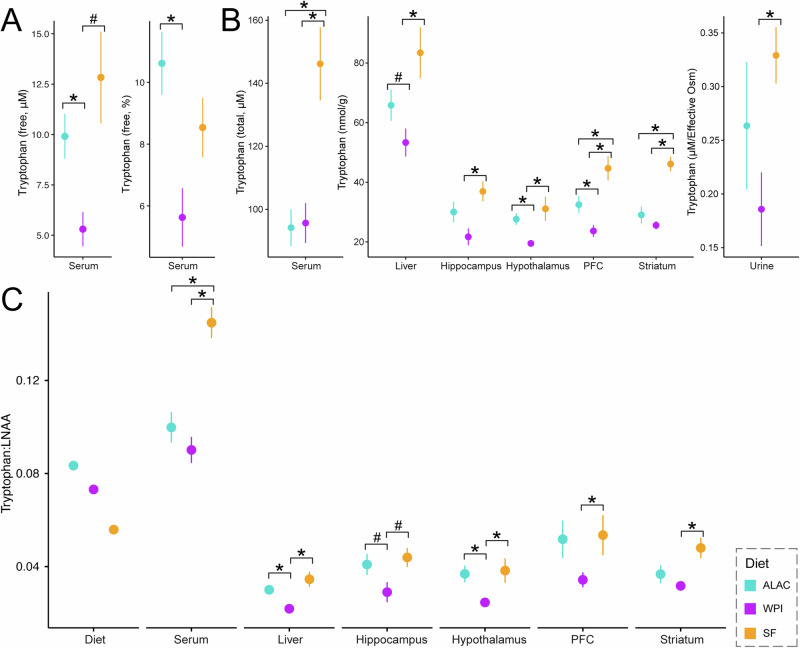
The impact of an α-lactalbumin-enriched formula on systemic tryptophan levels on Day 16. **A** Free tryptophan concentrations and percentage of free tryptophan in serum derived from arterial blood collected via cardiac puncture at necropsy. **B** Tryptophan levels in serum (from arterial blood), liver, brain, and urine. **A**, **B** Differences between the SF group and one of the formula-fed groups were evaluated using ANOVA followed by Tukey HSD post-hoc tests on generalized log-transformed data, accounting for litter effects in the model. Similarly, differences between the ALAC and WPI groups were assessed using ANOVA on generalized log-transformed data. For serum and liver data, both formula volume, postprandial time, and litter effect were included as factors in the model. For liver, brain, and urine, only the litter effect was accounted for. **C** Tryptophan to other large neutral amino acids (Tryptophan:LNAA) in the diet, serum, liver, and brain. Group differences were evaluated using a Mann–Whitney *U* test. Data are represented as mean ± SEM. *p < 0.05, #*p* < 0.1. PFC prefrontal cortex.

Furthermore, we evaluated the levels of tryptophan and the tryptophan:LNAA ratios across various biofluids and tissue compartments. In circulation, most tryptophan is albumin-bound, serving as a reservoir that releases free tryptophan as needed to ensure a sufficient supply of tryptophan for various physiological processes. Only a small fraction of tryptophan is free, which is the form available for organ uptake. We analyzed both free and total tryptophan in the serum and revealed that, on average, 8% of the circulating tryptophan is in the free form. The levels of free tryptophan, as well as the percentage of free tryptophan, were significantly higher in the ALAC piglets compared to WPI piglets (Fig. 3A). Contrary to results from Day 11 or 12, circulating total tryptophan levels measured on Day 16 were not significantly elevated in the ALAC group despite having higher tryptophan content in the diet (Fig. 3B). We speculate this discrepancy in tryptophan level may be due to different types of blood samples collected for the analysis as venous blood was used for measurements on Day 11 or 12, whereas arterial blood was taken via cardiac puncture at necropsy on Day 16. Generally, arterial concentrations of amino acids are higher than those in venous blood^31^, therefore, this may explain why the apparent tryptophan levels in the ALAC group were less pronounced on Day 16.

Nevertheless, compared to the WPI piglets, tryptophan or tryptophan:LNAA ratios were consistently higher in the liver and the brain of the ALAC piglets (Fig. 3B, C), indicating a systemic elevation of tryptophan availability for tissue uptake. Unexpectedly, the SF piglets, whose diet contains the least tryptophan, exhibited the highest circulating tryptophan levels and tryptophan:LNAA ratios in the serum, liver, brain, and urine. Notably, the differences in these measurements were less pronounced between the SF and ALAC piglets than between the SF and WPI piglets.

**Fig. 4:**
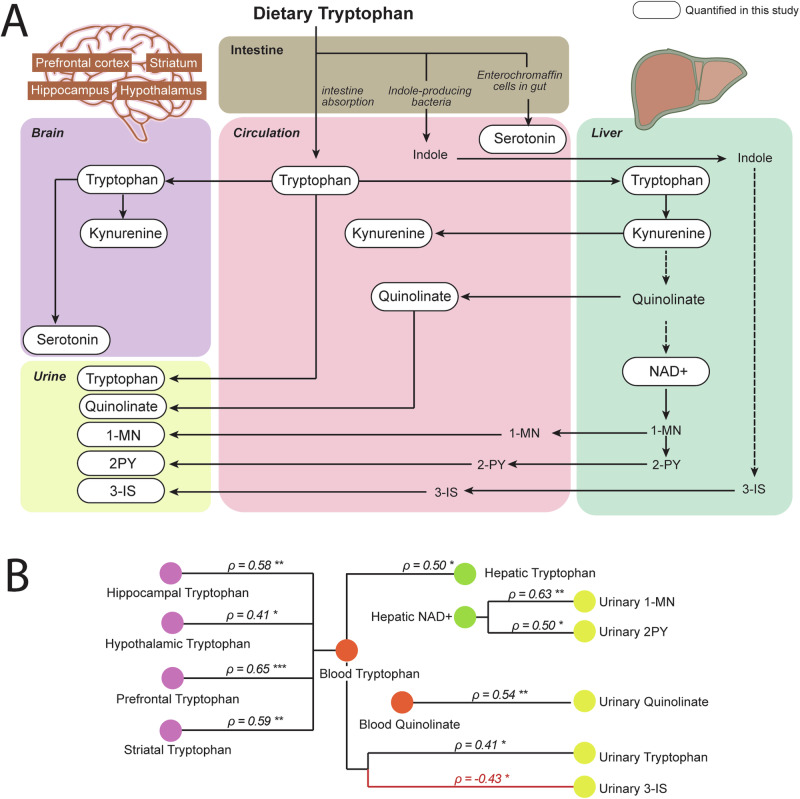
Metabolic pathways of tryptophan in key organs. **A** Following a diet enriched with tryptophan, there is an expected increase in circulating tryptophan levels. Most of the diet-derived tryptophan in circulation is shunted into the kynurenine pathway in the liver, where it ultimately contributes to de novo NAD+ synthesis. Intermediate metabolites of the kynurenine pathway, such as kynurenine and quinolinate, are released back into circulation. The end-products of the kynurenine pathway, including 1-methylnicotinamide (1-MN) and *N*-methyl-2-pyridone-5-carboxamide (2-PY), along with tryptophan, kynurenine, and quinolinate, are excreted in the urine. Some of the circulating tryptophan is taken up by the brain and utilized for serotonin synthesis via the enzyme tryptophan hydroxylase. Additionally, intestinal enterochromaffin cells also use tryptophan to produce serotonin, which significantly contributes to circulating serotonin levels. In the gut, indole-producing bacteria convert tryptophan into indole, which, upon absorption, is metabolized in the liver into 3-indoxyl sulfate (3-IS), with the majority subsequently excreted in the urine. **B** Correlation of tryptophan metabolites across various biofluids and tissue compartments illustrates the interconnected pathway of tryptophan metabolites within the body. Spearman correlation coefficient (*ρ*) was used to evaluate the strength of this relationship. Data from all three groups were used for the analysis. Multiple hypothesis testing was not adjusted. Only significant pairs are displayed. ***unadjusted *p* < 0.001, **unadjusted *p* < 0.01, *unadjusted *p* < 0.05.

Following a diet enriched with tryptophan, higher circulating tryptophan levels result in diverse metabolic responses. Most tryptophan is metabolized through the kynurenine pathway in the liver, while the brain and gut also convert tryptophan into serotonin, significantly influencing both cerebral and circulating serotonin levels. Excess tryptophan and catabolic products are primarily excreted in the urine. The metabolic fate of tryptophan is illustrated in Fig. 4A. To elucidate the metabolic flux between organs after feeding the α-lactalbumin-enriched formula, pairwise relationships between tryptophan and its catabolites were quantified and assessed using Pearson’s correlation. Our results reveal significant positive relationships between circulating tryptophan levels and tryptophan levels in the liver, brain, and urine. Circulating quinolinate levels showed positive correlations with urinary quinolinate. Additionally, hepatic NAD^+^ levels were positively associated with its downstream metabolic products, 1-methylnicotinamide (1-MN) and *N*-methyl-2-pyridone-5-carboxamide (2-PY), in the urine (Fig. 4B). Interestingly, circulating tryptophan levels negatively correlated with urinary 3-indoxyl sulfate (3-IS) (Fig. 4B), indicating an interaction between host tryptophan utilization and microbial conversion of tryptophan into indole, the precursor to 3-IS.

**Fig. 5:**
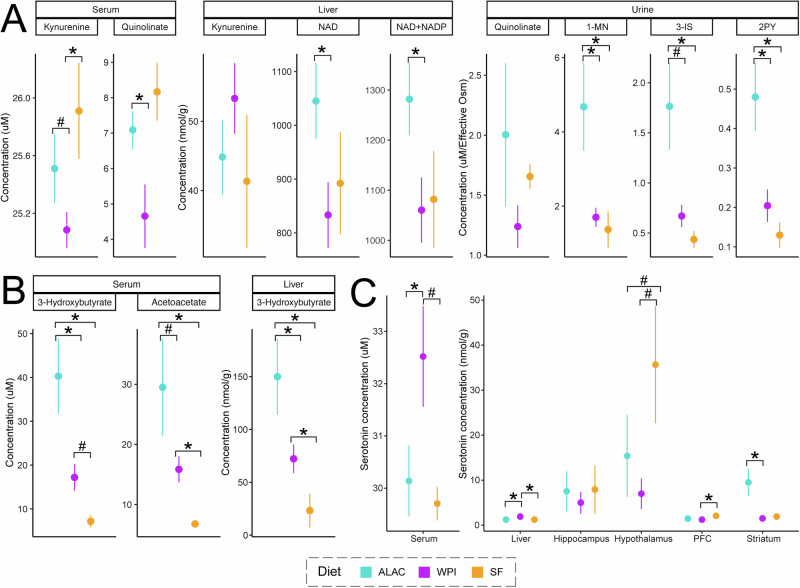
The impact of α-lactalbumin-enriched formula on the kynurenine pathway, ketogenesis, and serotonin levels on Day 16. **A** Levels of key metabolic products of the kynurenine pathway in serum, liver, and urine. **B** Concentrations of ketone bodies in serum and liver. **C** Serotonin levels in serum, liver, and 4 brain regions. **A**–**C** The differences between the SF group and one of the formula-fed groups were evaluated using ANOVA followed by Tukey HSD post-hoc tests on generalized log-transformed data, accounting for litter effects in the model. Similarly, the differences between the ALAC and WPI groups were assessed using ANOVA on generalized log-transformed data. For serum and liver data, both formula volume, postprandial time, and litter effect were included as factors in the model. For liver, brain, and urine, only the litter effect was accounted for. Data are represented as mean ± SEM. *p < 0.05, #*p* < 0.1. PFC prefrontal cortex.

Corresponding with the higher dietary tryptophan intake, ALAC piglets exhibited significantly higher levels of metabolic products from the kynurenine and indole pathways in their serum, liver, and urine (Fig. 5A). These findings regarding kynurenine metabolites partly align with observations made on Day 11 or 12 (Fig. 2D, E). Consequently, both the hepatic NAD^+^ and the summed concentrations of NAD^+^ and NADP^+^ were significantly higher in the ALAC piglets than in the WPI piglets. Correspondingly, higher levels of circulating and liver ketone bodies were observed in the ALAC piglets (Fig. 5B), suggesting a potential shift in energy state towards fat metabolism.

On Day 16, both the circulating and hepatic serotonin, which is primarily produced in the gastrointestinal tract, were significantly lower in the ALAC piglets than WPI piglets, aligning with the levels found in the SF piglets (Fig. 5C). Notably, the difference in postprandial circulating serotonin on Day 11 or 12 was not significant between the ALAC piglets and WPI piglets (Fig. 2F). This inconsistency might be due to the difference in age between when postprandial samples were taken and necropsy. Importantly, serotonin produced in the gut cannot cross the blood-brain barrier. In the brain, although tryptophan levels were similar across the four regions, serotonin concentrations were particularly higher in the hypothalamus. SF piglets tended to have the highest serotonin levels in the hypothalamus compared to the other two formula-fed groups. Notably, serotonin levels in the striatum were significantly higher in the ALAC piglets, highlighting enhanced serotonin synthesis in this region (Fig. 5C).

**Fig. 6:**
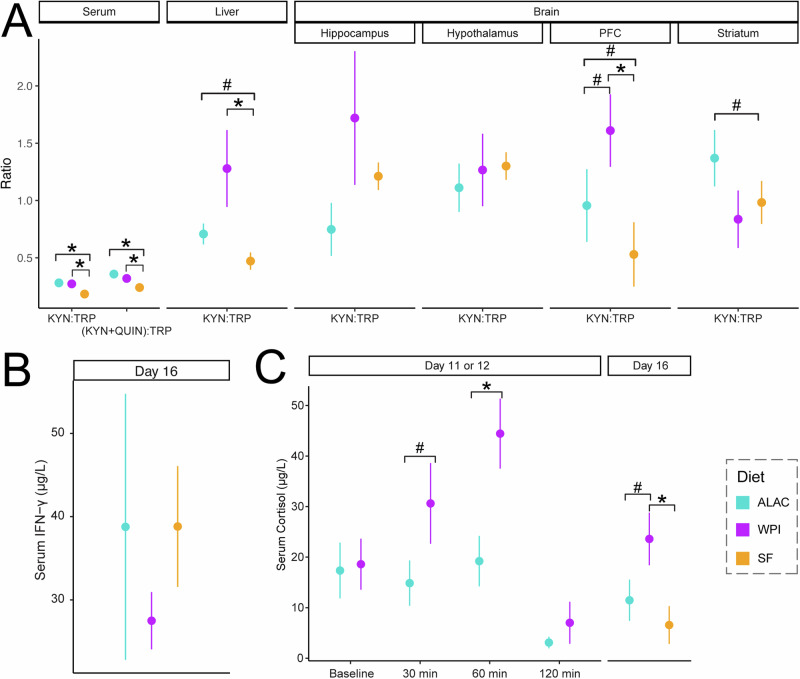
The impact of an α-lactalbumin-enriched formula on the conversion rate of tryptophan to kynurenine. **A** The ratio of kynurenine to tryptophan (KYN:TRP) and/or the sum of kynurenine and quinolinate to tryptophan ((KYN+QUIN):TRP) in serum, liver, and brain. The group differences were evaluated using the Mann–Whitney *U* test. **B** Serum IFN-γ concentration. The group difference was evaluated using ANOVA followed by Tukey HSD post-hoc test, with the litter effect accounted for in the model. **C** Serum cortisol levels at baseline, and at 30, 60, and 120 min post-meal intake on Day 11 or 12, as well as on Day 16. The group differences were evaluated cross-sectionally using the Mann–Whitney *U* test. Data are represented as mean ± SEM. *p < 0.05, #*p* < 0.1. PFC prefrontal cortex.

To evaluate the rate of conversion of tryptophan to kynurenine, we assessed two metabolite ratios: [KYN]:[TRP] (kynurenine to tryptophan) in serum, liver, and brain, and [KYN + QUIN]:[TRP] (kynurenine and quinolinate to tryptophan) in serum. In the serum, both formula-fed groups showed elevated [KYN]:[TRP] and [KYN + QUIN]:[TRP] ratios (Fig. 6A). In the liver and prefrontal cortex, the ratios of [KYN]:[TRP] was higher in the WPI piglets, significantly lower in the SF piglets, with the levels in ALAC piglets falling intermediate to the SF and WPI piglets (Fig. 6A). We further speculate a specific regulatory mechanism might drive these observations, which will be further explored in the next section.

### Unraveling diet-induced regulators of the tryptophan pathway from intake to liver and brain

The activities of the two rate-limiting enzymes of the kynurenine pathway, indoleamine 2,3-dioxygenase-1 (IDO-1) and tryptophan 2,3-dioxygenase (TDO), are essential for regulating the availability of tryptophan for serotonin biosynthesis in the brain. IDO-1, which is highly expressed in the intestinal tract and immune cells, is known to be transcriptionally regulated by the proinflammatory cytokine IFN-γ^32^. To assess the potential impact of IFN-γ levels on the kynurenine pathway, serum IFN-γ was assessed on Day 16. We found no significant differences in serum IFN-γ levels between the dietary groups (Fig. 6B), suggesting that the diet-dependent effect on the shift of tryptophan to the kynurenine pathway was not mediated by IFN-γ-induced immune activation.

In addition, we evaluated cortisol levels. Cortisol activates TDO, an enzyme primarily found in the liver and central nervous system^32^. Serum cortisol levels were measured on Days 11 or 12, and again on Day 16. We observed a postprandial increase in serum cortisol levels, with these levels tending to be higher in WPI piglets at 30 min, reaching statistical significance at 60 min post-meal compared to the ALAC piglets. This trend was also observed on Day 16, where cortisol levels remained significantly higher in the WPI piglets (Fig. 6C). These findings are consistent with the higher [KYN]:[TRP] ratio observed in the liver and prefrontal cortex of WPI piglets. The notably lower cortisol levels in ALAC piglets, comparable to those in the SF group, suggest that a diet-induced hormonal regulation might play a role in reducing activity of the kynurenine pathway, thereby preserving more tryptophan for serotonin biosynthesis in the brain.

## Discussion

α-Lactalbumin is a key component of human milk, yet typical cow’s milk-based infant formulas often contain lower levels of α-lactalbumin and higher levels of β-lactoglobulin. In this study, we utilized neonatal piglets as a translational model to assess the metabolic impacts of consuming a formula enriched with α-lactalbumin. Through detailed analysis of serum, urine, liver, and brain metabolites involved with tryptophan pathways, our study demonstrated that incorporating α-lactalbumin into the neonatal diet significantly enhanced the availability of tryptophan for developing infants. The enrichment of the tryptophan:LNAA ratio in the formula was directly reflected in circulating levels and subsequently influenced tryptophan uptake, particularly in the brain and liver. High dietary tryptophan led to elevated metabolites from both the kynurenine and indole pathways, which were predominantly excreted in the urine. Additionally, following a diet enriched with α-lactalbumin, we observed elevated hepatic NAD^+^ levels and ketone production, suggesting an impact on energy metabolism and a shift toward fat metabolism.

Sleep is essential for proper brain development in infants, and there is compelling evidence linking tryptophan intake to sleep quality. Previous research has demonstrated that formulas with higher levels of free tryptophan significantly increase circulating tryptophan and tryptophan:LNAA ratios and urinary markers of serotonin metabolism, leading to improvements in infant sleep patterns^33,34^. Although sleep patterns and quality were not directly evaluated in this study, our findings align with the previous observations, showing significantly higher hypothalamic and prefrontal tryptophan as well as increased striatal serotonin levels in piglets consuming the α-lactalbumin-enriched formula. In humans, research has consistently demonstrated higher tryptophan:LNAA ratio can be achieved by α-lactalbumin, influencing cognitive function, mood, and sleep^35^. Tryptophan serves as a precursor of serotonin, a neurotransmitter that is critical for cognitive functions such as mood, alertness, memory, attention, and executive function^36^. The metabolic downstream product of serotonin is melatonin, a hormone that regulates circadian rhythms and sleep patterns^37^, and both are essential for proper brain development. Additionally, piglets fed the α-lactalbumin-enriched formula showed reduced cortisol levels, a stress hormone released in a diurnal pattern influenced by sleep and circadian rhythms^38^. Notably, these piglets also demonstrated faster appetite recovery after postprandial blood draws. Excessive cortisol excretion has been associated with behavioral, mood, cognitive, and attention abnormalities in adult life^39^. In stress-vulnerable subjects, α-lactalbumin supplementation increased the circulating tryptophan:LNAA ratio, lowered salivary cortisol levels, and improved mood under stress^40^. However, extensive research is still needed to elucidate the mechanism involved.

Circulating tryptophan levels fluctuate considerably after meals, primarily managed by the activation of the kynurenine pathway and regulated by two key enzymes: TDO and IDO-1, influencing the availability of tryptophan for uptake into the brain. IDO-1’s activity is induced by proinflammatory cytokines released during immune activation or under pathological conditions. In contrast, TDO, primarily expressed in the liver, is sensitive to tryptophan influx and regulated by the stress hormone cortisol^32^. In this study, we assessed the potential dietary influence on the activity of IDO and TDO. Our findings reveal that neonatal piglets consuming a formula predominantly high in β-lactoglobulin, which contains lower tryptophan content compared to the α-lactalbumin-enriched formula, showed enhanced conversion of tryptophan to the kynurenine pathway. We speculate that the shift is driven by their elevated cortisol levels, which consequently lead to reduced serotonin synthesis in the brain. This mechanism could be particularly critical for infants from regions with a high burden of infectious diseases. Future research is needed to confirm whether the mechanisms described here have practical implications and can induce clinically relevant immediate or long-term health effects in growing infants.

In recognition of the essential role of tryptophan in infant development, the current guidelines for infant formula recommend using human milk protein composition as a reference to determine formula protein composition on the basis that this achieves optimal circulating tryptophan levels. The main consideration is that infant formula must provide at least an equal level of each amino acid as that provided by human milk^41^. Furthermore, recent research suggests that the current amino acid requirements may be underestimated; specifically, tryptophan levels need to be increased by ~30% over previous recommended values^42^. Cow’s milk protein contains lower tryptophan than human milk protein^24^, making tryptophan one of the limiting amino acids in formula development. To meet this minimum tryptophan content, formulas are typically made with a higher protein content than human milk. However, this increased protein intake during infancy has been linked to a higher risk of obesity later in life^7^. While adding free tryptophan can address this limitation, it results in a sharper postprandial fluctuation compared to more gradual kinetics achieved with α-lactalbumin. Therefore, building an infant formula around a tryptophan-rich protein source is preferable, as it more closely mimics the physiological profile of breast milk and allows for lower total protein content without compromising protein quality.

This study has several notable strengths. It is the first study that comprehensively quantified sow milk composition and subsequently developed two piglet formulas enriched in α-lactalbumin or β-lactoglobulin, closely aligned with the nutritional profile of sow milk. Additionally, the formulas were administered via handfeeding to the piglets, simulating the interactive parent-child feeding experience essential for cognitive development. However, there are several limitations worth acknowledging. Despite careful handling and interaction with the piglets, early separation from the sow was unavoidable. Adaptation to bottle-feeding and potential stress induced by repeated blood draws during postprandial measurements may have introduced confounding factors that could influence comparisons between sow-fed and formula-fed piglets. Moreover, the piglet formulas in this study were designed to match the protein content of sow milk, differing from typical human infant formulas, which generally contain higher protein levels than human milk. Although oxytocin was administered to the sow to stimulate milk letdown, the exact timing and milk intake by the piglets remain unknown, adding further complexity when evaluating outcomes between the formula-fed and sow-fed piglets. Additionally, sow milk naturally has higher tryptophan content than human milk (sow: 0.66 g/L (Supplementary Table 2), human: 0.20 g/L^1^); thus, such a difference between pig and human nutritional requirements may represent a limitation inherent to the animal model. Lastly, although this research provides insights into how tryptophan from α-lactalbumin is metabolized in key metabolic tissues, the exact uptake and distribution of tryptophan and its metabolic products remain to be further elucidated through future isotope labeling studies.

The current study elucidates the mechanistic action regarding the metabolic impact of α-lactalbumin enrichment in the infant diet, with a particular focus on how tryptophan is distributed, metabolized, and utilized by the brain and liver once it enters the circulatory system. We demonstrated that tryptophan derived from α-lactalbumin significantly influences systemic tryptophan metabolism and enhances tryptophan availability across the blood-brain barrier, favoring serotonin synthesis in the brain. Additionally, it is crucial to recognize the complex interplay between the host and gut microbiota. Tryptophan that is not absorbed in the intestine can serve as a substrate for gut microbiota, leading to the production of indole and its derivatives. While these results enhance our understanding of the metabolic effects of α-lactalbumin, the influence of these biochemical changes on behavioral and cognitive outcomes remains to be elucidated. A future clinical study is essential to fully decipher these functional implications.

## Methods

### Animals

Samples used for characterizing the nutritional content of sow milk were collected from a single 2-year-and-2-month-old Yorkshire × Landrace sow nursing her third litter. For the feeding trial, piglets were born to three sows: 2 of Yorkshire × Landrace lineage and 1 of Hampshire × Yorkshire × Landrace lineage. Piglets were used from birth to postnatal day (Day) 16, and sows were returned to the herd at the completion of the study. Sows and their piglets were kept together in farrowing pens from birth until Day 5 for the ALAC and WPI groups, and until Day 16 for the SF group. Upon initiation of bottle feeding, piglets in each formula group were housed together in a single farrowing pen equipped with heat lamps for warmth. The study included approximately equal numbers of male and female piglets from each litter.

### Ethical approval

Animal handling was approved by the University of California, Davis (UC Davis) Institutional Animal Care and Use Committee (IACUC protocol #22011). All experiments were performed in accordance with relevant guidelines and regulations, adhering to the UC Davis Swine Teaching and Research Center husbandry protocols.

### Characteristics of the diet

Sow milk samples were collected following oxytocin injection to stimulate milk letdown on postpartum Days 6, 11, and 14. Samples were transported on ice, frozen within 30 min of collection, and stored at -80 °C until analysis.

#### Total amino acid analysis

A pooled milk sample, containing 39.5% of the volume from Day 6, 5.4% from Day 11, and 55.1% from Day 14, as well as the ALAC and WPI formulas, was analyzed for total amino acids. Samples were hydrolyzed using 6 N HCl at 110 °C. To accurately measure cysteine and methionine, samples were treated with performic acid prior to acid hydrolysis to create acid-stable cysteic acid and methionine sulfone, respectively. Conversion of methionine was complete, while cysteine conversion exceeded 95%^43^. Tryptophan concentrations were determined by performing alkaline hydrolysis using 4.2 N NaOH. Amino acid quantification was performed using an L-8800 Hitachi amino acid analyzer (Hitachi High Technologies Corporation, Tokyo, Japan) equipped with ion-exchange chromatography using a sodium citrate buffer system at the UC Davis Molecular Structure Facility. The total amino acids were determined as the sum of quantified amino acids.

#### Sow milk preparation and metabolite analysis

The two sow milk samples collected on postpartum Days 6 and 14 were used to determine the concentration of free amino acids, organic acids, and sugars in sow milk. Two methods were employed for sample preparation. The first method involved extracting sow milk using the modified Folch method. The second method utilized an ultrafiltration technique to isolate milk metabolites. Metabolite concentrations were expressed in mg/L, with most measurements determined based on the modified Folch extraction method, except for 2′-fucosyllactose (2′-FL) and 3′-sialyllactose (3′-SL), which exhibited better recovery using the ultrafiltration method. Detailed descriptions of both methods are provided below under “Targeted NMR-based analysis of serum, urine, milk, brain, and liver metabolites.”

#### Mineral analysis

A pooled milk sample, containing equal volumes from Day 6 and Day 14, as well as the ALAC and WPI formulas, was analyzed for mineral composition using ICP-MS. Samples were subjected to acid hydrolysis using 5 mL concentrated trace metal grade HNO_3_ in a MARS6 microwave digestion system (CEM Corporation, NC, USA) equipped with CEM MARSXpress™ vessels ramped to 195 °C for 15 min, and maintained at that temperature for an additional 15 min with a maximum power setting of 1500 W. Method blanks were employed to ensure that the method was free of significant contamination, and digestion control standards were utilized to ensure analyte recoveries were within acceptable limits. After digestion, the samples were brought up to a final volume of 15 mL with Milli-Q water.

Quantitative analysis was performed using an Agilent 8900 ICP-MS Triple Quadrupole (Agilent Technologies, USA), optimized for sensitive and accurate mineral quantification. The system, equipped with a PFA inert kit and platinum cones, was tuned and calibrated prior to sample analysis. The instrument operated in MS/MS Nogas, H_2_, He, O_2_ using a 3-point peak pattern, with three replicates per injection and 50 sweeps per replicate. Samples, external calibration standards, check standards, and blanks were introduced via an Agilent SPS 4 Autosampler (Agilent Technologies, USA) using a 0.5 mm ID sample probe. These samples and standards were mixed at a ~17:1 ratio with a custom internal standard solution using a mixing tee. The mixed samples were then delivered to the ICP-MS via a peristaltic pump at a flow rate of 0.10 rps (revolutions per second). Aerosol generation was achieved by a 200 µL/min PFA concentric nebulizer in a 2 °C temperature-controlled double pass Scott-type PFA spray chamber, leading to a 1550 W plasma. To minimize polyatomic interference and ensure reliable measurements, collision/reaction cell gases (H_2_, He, O_2_) were utilized. Data processing was performed using MassHunter 4.6 software (version C.01.06, Agilent Technologies, USA).

### Formula production

Lactose and micellar casein concentrate were obtained from Leprino Foods Dairy Products, CO, USA. The α-lactalbumin-enriched whey (Lacprodan^®^ ALPHA-50) and standard whey protein isolate (Lacprodan^®^ DI-9224) were provided by Arla Foods Ingredients Group P/S, Denmark. Soybean oil, a refined, bleached, and deodorized product, was made by combining products from Columbus Vegetable Oils, IL, USA, and Kirkland Signature soybean oil. The mineral mix was made in-house and includes calcium phosphate monobasic monohydrate, calcium acetate monohydrate, potassium citrate monohydrate, potassium chloride, magnesium acetate tetrahydrate, zinc sulfate heptahydrate, manganese sulfate tetrahydrate, copper sulfate, potassium iodate, and sodium selenite sourced from Acros Organics, Alfa Aesar, Sigma, and Fisher. Free amino acids and essential nutrients used in formula development include L-arginine, L-glycine, L-histidine, L-methionine, L-proline, L-glutamate, taurine, and *myo*-inositol, which were sourced from manufacturers including Acros Organics, Alfa Aesar, Fisher, and Sigma.

The ALAC and WPI formulas were produced in the Milk Processing Laboratory at UC Davis. A total of 180 L of each formula was produced in two batches of 90 L each. Prior to the formula production, storage buckets and equipment were sanitized using F-29 liquid sanitizer (dodecyl dimethylammonium chloride solution, RMC, NY, USA). The vitamin mix, mineral mix, free amino acids, taurine, and *myo*-inositol were blended in distilled water at room temperature and added one at a time under continuous stirring. To prevent casein coagulation, the micronutrient mixture was pH adjusted to 6.2–6.6 using food-grade NaOH and KOH to neutralize the acidity of the vitamin mix and mineral mix while ensuring that the added amounts did not result in sodium and potassium levels exceeding those found in sow milk.

Lactose was dissolved in distilled water heated to 45 °C in a separate 25-gallon stainless steel tank. The whey, micronutrient mixture, and casein were added sequentially with constant stirring. Soybean oil was pre-emulsified with the protein mixture using a high-speed emulsion blender to achieve the desired consistency. The final formula product was adjusted to pH 6.3–6.4 if needed, and then processed through an indirect tubular continuous pasteurizer (Ultra High Temperature (UHT)/High Temperature Short Time (HTST) Lab 25 EHV Hybrid w/PLC, MicroThermics, Raleigh, NC, USA), followed by homogenization, and an aseptic filling hood. HTST pasteurization (72 °C for 15 s) was chosen over UHT (135 °C for 2–5 s) to avoid denaturation of α-lactalbumin^44^, and homogenization was performed at a flow rate of 2 L/min with the first-stage pressure of 13.8 MPa and a second-stage pressure of 1.38 MPa. The final formula products were stored at -20 °C in freezer-safe containers until use. To ensure food safety, food microbiological testing for the detection of pathogens was performed on each batch of formula. Samples collected from the aseptic filling hood (one from each batch) were sent to Deibel Laboratory (Manteca, CA) for detection of *Salmonella*, *E. coli*, *Listeria* sp, *Bacillus cereus*, *Campylobacter*, Enterobacteriaceae, mesophilic aerobic spores, yeast, mold, and total anaerobic counts.

### Animal study

A total of 30 piglets from 3 sows were allowed to remain with their sows until Day 5 to ensure proper immune development. On Day 3, all piglets received 200 mg of elemental iron as iron dextran, and male piglets were castrated. On Day 5, piglets were sex-, weight-, and litter-matched, and then randomized and assigned into one of three dietary groups. The ALAC group (*n* = 12, 6 males and 6 females) received a formula enriched with α-lactalbumin (Lacprodan^®^ ALPHA-50, provided by Arla Foods Ingredients Group P/S, Denmark). The WPI group (*n* = 12, 6 males and 6 females) received a formula made with standard whey protein isolate that is primarily β-lactoglobulin but low in α-lactalbumin (Lacprodan^®^ DI-9224, provided by Arla Foods Ingredients Group P/S, Denmark). The sow-fed (SF) reference group (*n* = 6, 3 males and 3 females) continued suckling from their birth sows. The composition of the ALAC and WPI formulas is provided in Supplementary Tables 2–4. On Day 7, one SF piglet was euthanized due to an accidental injury caused by the sow. Additionally, one female piglet from the ALAC group was reassigned to the SF group due to repeated attempts to return to her sow and unwillingness to consume the assigned formula. This resulted in a total of 11 ALAC (6 males, 5 females), 12 WPI piglets (6 males, 6 females), and 6 SF piglets (3 males, 3 females). Body weight was recorded daily to monitor growth and determine daily formula requirements.

To support normal social development and minimize separation stress, ALAC and WPI piglets were group-housed in two farrowing pens and received their respective formulas throughout the feeding intervention. The volume of formula provided to the ALAC and WPI piglets was determined daily to meet their caloric needs and administered in 8 equal portions every 3 h. Formula intake was recorded at each meal to determine daily formula intake.

On Days 11 and 12, piglets from the ALAC and WPI formula groups underwent postprandial blood draws from the cephalic vein, with half of the piglets in each group sampled each day. Piglets were fasted for 5 h prior to baseline blood sample collection. They were then allowed to consume up to 120 mL of their assigned formula within a 15-min window. After feeding, the piglets were returned to their respective pens, and subsequent blood samples were collected at 30, 60, and 120 min post-meal.

Prior to euthanasia, piglets in the ALAC and WPI groups were fasted for ~5 h, and then allowed to consume up to 120 mL of formula over 15 min. Formula-fed piglets were anesthetized ~1 h post-meal. To achieve a similar postprandial time in the SF piglets, the corresponding sows were injected with 20 USP units of oxytocin 90 min prior to sacrifice to stimulate milk let down ~1 h prior to sacrifice. However, the precise feeding time and intake volume of the sow milk by each piglet could not be assessed.

All piglets were anesthetized using a premixed solution containing Telazol™ (100 mg/mL), ketamine (50 mg/mL), and xylazine (50 mg/mL) at a dosage of 0.04 mg/kg. Blood samples were collected via cardiac puncture, and piglets were subsequently euthanized using pentobarbital (FatalPlus™, Vortech Pharmaceuticals, Dearborn, MI) at a dosage of 0.22 mL/kg. Immediately following euthanasia, urine was collected through bladder puncture, although samples from 3 piglets (1 ALAC, 2 WPI) could not be obtained. The brain, liver, and kidneys were weighed. Specific brain regions (hippocampus, striatum, prefrontal cortex, and hypothalamus) and a section of the left lateral lobe of the liver were collected, snap-frozen in liquid nitrogen, and stored at -80 °C until further analysis.

### Hemoglobin and hematocrit measurements

Hemoglobin and hematocrit were assessed on heparinized whole blood at sacrifice. Hemoglobin concentrations were measured using Drabkin’s reagent (cyanmethemoglobin method) in duplicate according to the manufacturer’s instructions (Sigma Aldrich, St. Louis, MO). Hematocrit levels were assessed by first filling a heparinized microhematocrit capillary tube (Fisher Scientific, Hampton, NH) with ~65 µL of whole blood. This tube was then centrifuged at 15,000 × *g* for 5 min at room temperature. The resulting hematocrit values were determined using a microcapillary reader (Damon, Needham Heights, MA).

### Serum hormonal measurements

Serum concentrations of cortisol, insulin, ghrelin (acylated and desacylated), GLP-1 (7-36 and 7-37) were simultaneously quantified using a liquid chromatography-tandem mass spectrometry (LC-MS/MS) approach, as described by Zhang et al.^45^. The concentration of GLP-1 was determined as the sum of 7-36 and 7-37.

#### Standards and calibration curve

Standards for human ghrelin (acylated and desacylated) were obtained from R&D Systems (Minneapolis, MN, USA). Human and pig insulin, d4-cortisol, and cortisol were obtained from Millipore Sigma (Burlington, MA, USA). Pig GLP-1 (7-36 and 7-37, both methylated and unmethylated) and ghrelin (acylated and desacylated) were custom-synthesized by GeneScript (Piscataway, NJ, USA). All peptides had purities above 95% and were reconstituted according to the manufacturer’s specifications, diluted to the appropriate concentrations, and stored in aliquots at -20 °C until use.

The external standard mixture included pig insulin, pig ghrelin (acylated and desacylated), pig unmethylated GLP-1 (7-36 and 7-37), and cortisol. The internal standard mixture constituted 0.125 ng/mL for human insulin, human acylated ghrelin, human desacylated ghrelin, and pig methyl-GLP-1 (7-37); 0.187 ng/mL for pig methyl-GLP-1 (7-36); and 12.5 ng/mL for cortisol-d4. An eleven-point calibration curve was established at fixed concentrations of external standards and prepared with a constant concentration of internal standards. Calibration matrices were prepared by combining filtered and unfiltered piglet serum at a 40:60 ratio.

#### Sample preparation

Seven microliters of the internal standard mixture were spiked into 100 μL of piglet serum. Protein precipitation was then performed by mixing with a cold solvent (75% acetonitrile in water) with 0.2% acetic acid in a 1:4 ratio, followed by incubation at 4 °C for 30 min and centrifugation at 10,000 × *g* for 5 min. The supernatant was then filtered through a 0.2 µm polyvinylidene fluoride (PVDF) syringe filter prior to loading into QuanRecovery LC-MS vials. Quality control samples were prepared in duplicate by spiking 100 μL of the calibration matrix with 1 μL of standard stock solutions containing various concentrations of pig hormones. All reagents were of ACS grade. Low-protein-binding microcentrifuge tubes (Thermo Fisher Scientific, Waltham, MA, USA) were used to minimize non-specific binding of the peptide hormones.

#### LC-MS/MS analysis

Analysis was performed using an Agilent 1260 Infinity II LC system coupled with an Agilent 6470 Triple Quad LC/MS equipped with an electrospray ionization (ESI) source, operating in positive ion mode. Chromatographic separation was achieved on an Agilent AdvanceBio Peptide C18 column (120 Å, 2.1 × 150 mm, 2.7 µm). The mobile phases consisted of Milli-Q water with 0.2% formic acid (solvent A) and acetonitrile with 0.2% formic acid (solvent B). The column temperature was held at 50 °C, and the autosampler was set to 8 °C. Mass spectrometry setting was optimized as follows: gas temperature at 330 °C, gas flow at 10 L/min, nebulizer pressure at 30 psi, sheath gas temp at 350 °C, sheath gas flow at 11 L/min, capillary voltage at 3350 V, nozzle voltage at 500 V, and chamber current at 0.19 µA. An injection volume of 5 μL was used, and needle washing was performed with 100% LC-MS grade methanol. Dynamic multiple reaction monitoring was employed to ensure precise quantification.

Quantification was achieved using Agilent MassHunter software (version 10.1). The transitions that generated the highest and second-highest signal abundances were selected as quantifiers and qualifiers, respectively. The reliability of these transitions was verified using pig serum spiked with standards. The quantifier-to-qualifier ratios between the calibrators and samples were maintained for targeted quantification. A signal-to-noise ratio (S:N) of 5:1 was set for quantification. For signals falling below the limit of detection, values were approximated as the limit of quantification (LOQ) divided by the square root of 2, where LOQ represents the lowest spiked calibration point. The LOQ values established for each hormone were as follows: insulin at 4.9 pg/mL, acylated and desacylated ghrelin at 1.2 pg/mL each, GLP-1 (7-36 and 7-37) at 2.4 pg/mL, and cortisol at 0.12 ng/mL.

### Metabolite quantification across various biofluids and tissues

Tryptophan, kynurenine, and serotonin were quantified in serum, liver, and brain using targeted GC-MS. This method specifically assesses the total concentration, inclusive of both free and albumin-bound tryptophan. Additionally, ^1^H-NMR was used to quantify free tryptophan and quinolinate in serum, as well as tryptophan, quinolinate, 1-methylnicotinamide, *N*-methyl-2-pyridone-5-carboxamide, and 3-indoxyl sulfate in urine. Additionally, hepatic levels of NAD^+^ and NADP^+^ were determined using the modified Folch extraction method, also quantified via ^1^H-NMR. NMR spectral peak assignments for targeted metabolites in serum, urine, and liver are detailed in Supplementary Fig. 7.

#### Serum preparation from whole blood

Blood samples were collected in additive-free serum tubes, allowed to sit at room temperature for 30 min, and then centrifuged at 1600 × *g* at 4 °C for 15 min. The separated serum layer was subsequently collected and stored at -80 °C.

#### Untargeted and Targeted GC-MS-based analysis of serum, liver, and brain metabolites

Postprandial serum samples from Day 11 or 12 were assessed using untargeted GS-MS analysis, and serum, liver, and brain concentrations of tryptophan, kynurenine, and serotonin were quantified using targeted GS-MS analysis. Liver and brain tissues were manually ground in liquid nitrogen using a mortar and pestle before being submitted for analysis. Both untargeted and targeted data were performed by the West Coast Metabolomics Center (University of California Davis, Davis, CA), following a previously established protocol^46^. Briefly, samples were extracted using 1 mL of a solvent mixture composed of acetonitrile, isopropanol, and water in a 3:3:2 ratio (v/v/v). Each sample was divided into two equal aliquots. One aliquot was completely dried and then derivatized using 10 µL of 40 mg/mL methoxyamine in pyridine, and shaken for 90 min at 30 °C. A mixture of internal standards was added for quantification purposes, followed by 91 µL of *N*-Methyl-N-(trimethylsilyl)trifluoroacetamide (MSTFA) and an additional 30 min of shaking for 30 min at 37 °C to complete the derivatization.

Data were collected using a 7890 Agilent GC (Agilent Technologies, Santa Clara, CA) coupled to a Leco Pegasus IV TOFMS (Leco, St. Joseph, MI). A 0.5 µL of derivatized sample was injected using a splitless method onto an RTX-5SIL MS column with an Intergra-Guard (Restek, Centre County, PA) at 275 °C with a helium flow of 1 mL/min. The oven temperature was set to hold at 50 °C for 1 min, ramped to 300 °C at 20 °C/min, then held for 5 min. The ion source temperature was 250 °C, and mass spectral acquisitions were collected from 80 *m*/*z* to 500 *m*/*z* at a rate of 17 spectra/s. For targeted data, a calibration curve was injected from 0.1 to 20 µg/mL, which were acquired alongside the samples. Serum tryptophan, kynurenine, and serotonin were expressed in μmol/L (μM). Liver and brain tryptophan, kynurenine, and serotonin were expressed in nmol/g.

#### Targeted NMR-based analysis of serum, urine, milk, brain, and liver metabolites

Sow milk, serum, and urine samples were filtered through Amicon Ultra-0.5 mL centrifugal filters with a 3000 MW cutoff (Millipore, Billerica, MA) to remove proteins and lipids. To each 207 μL of filtrate, 23 μL of an internal standard solution was added, which contained 4.608 mM 3-(trimethylsilyl)-1-propanesulfonic acid-d6 (DSS-d6), 0.2% sodium azide (NaN_3_), and 99.8% deuterium oxide (D_2_O). The pH of each sample was then adjusted to 6.85 ± 0.1. Finally, 180 μL of the prepared sample was transferred to a 3 mm NMR tube (Bruker, Billerica, MA) and stored at 4 °C until spectral acquisition.

Sow milk, liver, and brain tissues were extracted using a modified Folch extraction described in Hasegawa et al.^47^. Initially, liver and brain tissues were manually ground in liquid nitrogen using a mortar and pestle. Then, ~30 mg of ground brain tissues, 75 mg of ground hepatic tissue, or 500 μL of sow milk were first mixed with a chloroform:methanol solution (CHCl_3_:MeOH, 2:1 v/v) containing 0.002% butylated hydroxytoluene (12.5:1 tissue-to-volume ratio), as well as 1 mM EDTA containing 0.9% w/v potassium chloride (50:1 tissue-to-volume ratio). The mixtures were vortexed, centrifuged, and the polar layer was collected. Samples were extracted a second time by mixing with another chloroform:methanol solution (CHCl_3_:MeOH, 10:1 v/v) at a 21.4:1 tissue-to-volume ratio. Samples were vortexed and the polar layer was collected and combined with that of the first extraction. The combined polar layer was then dried using a MiVac Duo Concentrator (Genevac Ltd, Ipswich, United Kingdom) and reconstituted in 100 mM phosphate buffer in D_2_O (adjusted to pH 6.86-6.91, if necessary). 207 µL of the reconstituted sample was combined with 23 µL of the internal standard, 3-(trimethylsilyl)-1-propanesulfonic acid-d_6_ (DSS-d6), containing 0.2% NaN_3_, and 99.8% D_2_O. Finally, a total volume of 180 μL was transferred to a 3 mm NMR tube (Bruker, Billerica, MA) and stored at 4 °C until spectral acquisition.

All ^1^H-NMR spectra were collected at 25 °C using the noseypr1d pulse sequence on a Bruker Avance 600 MHz spectrometer (Bruker, Billerica, MA) as described^5^. The NMR spectra were processed and analyzed using Chenomx NMR Suite (version 8.6, Chenomx, Edmonton, AB). Each spectrum was Fourier transformed, and then manually phase and baseline corrected in Processor. Metabolites were quantified by the same researcher in Profiler, using the concentration of the internal standard (DSS-d6) as reference.

Serum metabolites are expressed as μmol/L (μM). The concentrations of brain and liver metabolites were expressed in nmol/g wet weight. Urine metabolites were normalized by effective osmolality and expressed in μM/Osm. Urine osmolality was measured in duplicate using a VAPRO vapor pressure osmometer (ELITech, Logan, UT). Although urinary urea contributes to measured urine osmolality, since it freely crosses cell membranes, therefore, it does not contribute to tonicity. The effect of osmolality of each urine sample was determined by subtracting the molar contribution of urinary urea from the measured osmolality.

### Serum INF-γ measurement

Serum INF-γ levels were assessed in a subset of samples collected at necropsy (SF, *n* = 4; ALAC, *n* = 10; WPI, *n* = 10). Measurements were performed in duplicate using the Milliplex Map Porcine Cytokine/Chemokine multiplex assay (Millipore Sigma, Burlington, MA) according to the manufacturer’s instructions. The results were determined as the median of 100 beads, utilizing the Luminex 100^TM^ bioassay detection system equipped with xPONENT software (Luminex Co., Austin, TX).

### Statistical analysis

Statistical analysis and graphical representations were conducted using the R programming environment. Plots were generated utilizing the *ggplot* or *ggpubr* packages. Weight data were analyzed either cross-sectionally at enrollment (postnatal Day 4) or over the entire study period (postnatal Days 5–15) using ANOVA, followed by post-hoc Tukey HSD tests, adjusting for litter effect. Formula intake, hemoglobin, hematocrit, organ weight, and serum IFN-γ were evaluated using ANOVA with adjustment for litter effects. Differences in serum hormones and ratios of tryptophan to LNAA, kynurenine to tryptophan, and the sum of kynurenine and quinolinate to tryptophan were evaluated cross-sectionally using the Mann–Whitney *U* test.

For metabolite data, a generalized log transformation (defined as *log*(*y* + *sqrt*(*y*^2^ + *lambda*))) was applied to all metabolite data, where lambda is 1. Principal component analysis of the untargeted serum metabolomes from postprandial measurements on Day 11 or 12, as well as targeted quantification of tryptophan pathway metabolites in serum, urine, liver, and brain on Day 16, was performed using generalized log-transformed data to visualize dietary group differences.

For the postprandial serum metabolite concentrations on Days 11 and 12, group differences were evaluated cross-sectionally using ANOVA on the generalized log-transformed data, with litter effects accounted for in the model. For the Day 16 metabolite concentrations, differences between the SF group and one of the formula-fed groups were assessed using ANOVA followed by post-hoc Tukey HSD tests on generalized log-transformed data, adjusting for litter effects. Similarly, differences between the ALAC and WPI groups were assessed using ANOVA on generalized log-transformed data. Specifically for the serum and liver metabolites, formula volume, postprandial time, and litter effect were included as factors in the model, while for the liver, brain, and urine metabolites, only litter effect was accounted for in the ANOVA model. Both Pearson’s (*r*) and Spearman’s (*ρ*) correlation coefficients were employed to evaluate the strength of correlations. A significance level of *p* < 0.05 was considered statistically significant for all analyses.

## Supplementary information

Supplementary Information

## Data Availability

Data are available at xuahe.github.io/piglet-aLac-tryptophan-study.

## Code availability

The R scripts are available at xuahe.github.io/piglet-aLac-tryptophan-study.
