# Supplementary material for: The role of alpha-lactalbumin in modulating tryptophan metabolism and serotonin synthesis
Source: NPJ Sci Food. 2025 Jul 3;9:120. doi: 10.1038/s41538-025-00497-6 (PMC12229548; doi:10.1038/s41538-025-00497-6)
Supplement: Supplementary file 1 — Supplementary Information [file 41538_2025_497_MOESM1_ESM.pdf]

Supplementary information

The role of alpha-lactalbumin in modulating tryptophan metabolism and serotonin synthesis

Shannon Shoff, Xuan He, Hanna Lee, Zhichao Zhang, Darya O. Mishchuk, Gulustan Ozturk, Daniela Barile, Merete Lindberg Hartvigsen, Anne Staudt Kvistgaard, Carolyn M. Slupsky

Table of Contents

1. **Supplementary Tables**

- **Supplementary Table 1.** Metabolite concentrations in sow milk collected at postpartum day 6 and 14
- **Supplementary Table 2.** Nutrient composition of the ALAC formula, the WPI formula and sow milk
- **Supplementary Table 3.** Mineral composition of ALAC formula, WPI formula and sow milk
- **Supplementary Table 4.** Vitamin composition of ALAC formula, WPI formula and sow milk
- **Supplementary Table 5.** Organ weight by treatment group.

2. **Supplementary Figures**

- **Supplementary Fig 1.** Growth and formula intake
- **Supplementary Fig 2.** Assessment of hematocrit (Hct) and hemoglobin (Hb) on Day 16.
- **Supplementary Fig 3.** The impact of an α-lactalbumin enriched formula on circulating hormones.
- **Supplementary Fig 4.** The impact of α-lactalbumin on the overall postprandial serum metabolome.
- **Supplementary Fig 5.** Postprandial levels of tryptophan following consumption of formulas predominantly containing α-lactalbumin or β-lactoglobulin as major whey proteins.
- **Supplementary Fig 6.** Principal component analysis of serum, urine, liver and brain metabolomes on Day 16.
- **Supplementary Fig 7.** Representative 600 MHz ^{1}H-NMR spectra from serum, urine and liver samples showing peak assignments for tryptophan metabolism-related metabolites quantified in this study.

Supplementary Table 1. Metabolite concentrations in sow milk collected at postpartum day 6 and 14.

Milk Metabolite	Postpartum day 6	Postpartum day 14	Mean ± SD
2'-Fucosyllactose	63.9	49.5	56.7 ± 10.2
3'-Sialyllactose	596.1	276.8	436.4 ± 225.8
AMP	33.7	22.7	28.2 ± 7.8
Acetate	4.5	5.3	4.9 ± 0.6
Choline	2.2	2.1	2.2 ± 0.1
Citrate	1,542.60	1,504.50	1,523.6 ± 27.0
Creatine	111.5	134.4	123.0 ± 16.2
Creatine phosphate	185.4	186.5	185.9 ± 0.8
Formate	1	1.9	1.4 ± 0.7
Glycerophosphocholine	339.8	353.8	346.8 ± 9.9
Isoleucine	1.3	4.2	2.7 ± 2.0
Lactate	17.1	20.6	18.9 ± 2.5
Lacto-n-neotetraose	223.1	109.9	166.5 ± 80.0
Lactose	46,647.90	62,520.00	54,583.9 ± 11,223.2
Leucine	6.8	5.1	5.9 ± 1.3
Lysine	15.3	18.3	16.8 ± 2.1
Methanol	96	165.6	130.8 ± 49.2
Methionine	10.3	7.6	8.9 ± 1.9
N-Acetylglutamine	150.1	102.1	126.1 ± 33.9
NAD^+	n.d.	23.6	11.8 ± 16.7
O-Phosphocholine	435.5	431.6	433.6 ± 2.8
Taurine	159.9	171.9	165.9 ± 8.5
Threonine	6.7	25.6	16.2 ± 13.3
UDP-N-Acetylglucosamine	272.6	88.4	180.5 ± 130.3
UDP-galactose	839.2	465.8	652.5 ± 264.0
UDP-glucose	218.5	214.3	216.4 ± 3.0
UMP	336.5	152.1	244.3 ± 130.4
Uracil	0.1	0.8	0.4 ± 0.5
myo-Inositol	501.7	395.6	448.7 ± 75.0

Metabolite concentrations expressed in mg/L. For amino acids, the concentration represents free amino acids.
Abbreviations: AMP, adenosine monophosphate; NAD^+, nicotinamide adenine dinucleotide; UDP, uridine diphosphate; UMP, uridine monophosphate.

Supplementary Table 2. Nutrient composition of the ALAC formula, the WPI formula and sow milk.

	ALAC formula<br>*formula made with α-lactalbumin enriched whey protein isolate*	WPI formula<br>*formula made with standard whey protein isolate*	Sow milk<br>*Quantified using pooled sow milk*	 *Literature values*
Energy (kcal/L)	1068.2	1063.2		1140[1]
Carbohydrate *(g/L)*	56.55	56.55		
Lactose (g/L)	*55.00*	*55.00*	*54.5*	*54[1]; 41.1-55.3[2]*
Corn starch (g/L)	*1.55*	*1.55*		
Protein (g/L)	44.30 ± 0.85	43.15 ± 0.78	46.19	42.2[1]; 48.3-50.4[2]; 54[3]
Whey:casein ratio	*49.5:50.5*	*50:50*		*50:50[4]*
α-Lactalbumin (g/L)	*18.33*	*1.83*		
β-Lactoglobulin (g/L)	*1.43*	*12.94*		
Casein glycomacropeptide (g/L)	*0.00*	*4.25*		
Fat (g/L)	70.00	70.00		71[1]; 73.3-78.4[2] ; 76[3]
Amino acid composition (g/L)				
Alanine	1.00 ± 0.00	1.66 ± 0.03	1.52	2.47[1]
Arginine	1.65 ± 0.04	1.68 ± 0.01	2.13	0.89[1]
Arg from protein	*0.72 ± 0.04*	*0.97 ± 0.01*		
Addition of free Arg	*0.93*	*0.70*		
Aspartate/asparagine	4.89 ± 0.08	3.62 ± 0.05	3.86	4.21[1]
Methionine	1.00 ± 0.04	1.12 ± 0.00	1.07	0.74[1]
Met from protein	*0.81 ± 0.04*	*1.12 ± 0.00*		
Addition of free Met	*0.20*	*0.00*		
Cysteine	1.16 ± 0.05	0.62 ± 0.01	0.68	0.65[1]
Glutamate/glutamine	7.80 ± 0.23	8.01 ± 0.07	9.21	6.83[1]
Glu/Gln from protein	*6.85 ± 0.23*	*8.01 ± 0.07*		
Addition of free Glu	*0.95*	*0.00*		
Glycine	0.89 ± 0.01	0.88	1.18	1.64[1]
Gly from protein	*0.78 ± 0.01*	*0.43*		
Addition of free Gly	*0.11*	*0.45*		
Histidine	1.09 ± 0.00	0.97	1.32	0.65[1]
His from protein	*1.09 ± 0.00*	*0.77*		
Addition of free His	*0.00*	*0.20*		
Isoleucine	2.24 ± 0.04	2.39 ± 0.08	1.96	1.94[1]
Leucine	4.07 ± 0.08	4.11 ± 0.08	3.79	3.71[1]
Lysine	3.89 ± 0.03	3.62 ± 0.04	3.60	3.10[1]
Phenylalanine	1.89 ± 0.06	1.59 ± 0.01	1.81	1.40[1]
Tyrosine	1.98 ± 0.05	1.64 ± 0.00	1.95	1.23[1]
Proline	3.70 ± 0.06	3.74 ± 0.04	4.71	5.33[1]
Pro from protein	*1.99 ± 0.06*	*2.97 ± 0.04*		
Addition of free Pro	*1.71*	*0.77*		
Serine	1.97 ± 0.05	2.03 ± 0.01	2.36	2.56[1]
Threonine	1.95 ± 0.01	2.35 ± 0.02	2.08	2.15[1]
Tryptophan	1.03 ± 0.05	0.89 ± 0.10	0.66	0.35[1]
Valine	2.12 ± 0.04	2.44 ± 0.03	2.30	2.39[1]
Taurine	166.00	166.00	165.9	

Nutrient composition of each piglet formula was based on amounts and purity of ingredients used in production, as well as results from total amino acid analysis. After diet production, total amino acids (except glycine and histidine from the WPI

formula) were measured from two production batches. The concentration of glycine and histidine from the WPI formula were determined from a single replicate. Amino acid results are expressed in mean ± standard deviation. Concentration of lactose, *α*-lactalbumin*, β*-lactoglobulin, casein glycomacropeptide, and free amino acids in the pig formulas were estimated based on ingredient product information and added quantity.

Total amino acids in sow milk were measured using a pooled milk sample containing 39.5% of the volume from postpartum day 6, 5.4% from postpartum day 11, and 55.1% from postpartum day 14. The total protein concentration of measured sow milk was as determined as the sum of total amino acids. Lactose in sow milk was quantified using ^{1}H-NMR and determined as the average of measurements from postpartum day 6 and 14.

Supplementary Table 3. Mineral composition of ALAC formula, WPI formula and sow milk.

	**ALAC formula**<br>*formula made with α-lactalbumin enriched whey protein isolate*	**WPI formula**<br>*formula made with standard whey protein isolate*	**Sow milk**<br>*Quantified using pooled sow milk*
Macro-minerals (mg/dL)			
Calcium	208	193	207.7
Phosphorus	130	118	165.6
Potassium	143	152	103.6
Chloride	102	93	36.9
Sodium	55	54	34.1
Micro-minerals (mg/L)			
Magnesium	120	120	116
Iodine	40	40	not measured
Zinc	8.2	7.2	7.83
Manganese	0.3	0.3	0.027
Copper	1.4	1.3	1.73
Iron	0.6	0.7	0.88
Selenium	0.1	0.1	0.113

Values were determined by direct measurement of mineral composition of a pooled milk sample, containing 50% of the volume from postpartum day 6 and 50% from postpartum day 14, as well as the ALAC and WPI formulas using inductively coupled plasma mass spectrometry (ICP-MS). The measurement of chloride may be underestimated due to background noise.

Supplementary Table 4. Vitamin composition of ALAC formula, WPI formula and sow milk.

	**ALAC formula** *formula made with α-lactalbumin enriched whey protein isolate*	**WPI formula** *formula made with standard whey protein isolate*	**Sow milk**	
			Quantified using pooled sow milk	*Literature values*
Vitamin A (IU/L)	1754.42	1754.82		466.67-2433.33[3]
Thiamin (mg/L)	1.58	1.58		0.68-0.8[3]
Riboflavin (mg/L)	1.95	1.95		0.46-2.1[3]
Niacin (mg/L)	8.77	8.77		No data
Pantothenate (mg/L)	5.36	5.36		No data
Vitamin B6 (mg/L)	1.6	1.6		0.101[3]
Biotin (mg/L)	0.04	0.04		0.024-0.068[3]
Inositol (mg/L)	436.75	436.75	448.7	
Folic acid (mg/L)	0.18	0.18		0.0023-0.134[3]
Vitamin B12 (mg/L)	0.0026	0.0026		0.00241[3]
Choline (mg/L)	124.41	124.44	2.2	
Vitamin C (mg/L)	87.72	87.74		45-130[3]
Vitamin D (IU/L)	194.92	194.97		120-360[3]
Vitamin E (IU/L)	10.72	10.72		1.79-5.82[3]
Vitamin K (mg/L)	4.39	4.39		0.089-0.101[3]

Choline and inositol levels in sow milk were quantified using ^{1}H NMR and determined as the average of measurements from postpartum day 6 and 14.

References

1. Odle, J., Lin, X., Jacobi, S.K., Kim, S.W., and Stahl, C.H. (2014). The Suckling Piglet as an Agrimedical Model for the Study of Pediatric Nutrition and Metabolism. Annu. Rev. Anim. Biosci. *2*, 419–444. https://doi.org/10.1146/annurev-animal-022513-114158.

2. Zhang, S., Chen, F., Zhang, Y., Lv, Y., Heng, J., Min, T., Li, L., and Guan, W. (2018). Recent progress of porcine milk components and mammary gland function. J. Anim. Sci. Biotechnol. *9*, 77. https://doi.org/10.1186/s40104-018-0291-8.

3. Hurley, W.L. (2015). Composition of sow colostrum and milk. In The gestating and lactating sow, C. Farmer, ed. (Wageningen Academic Publishers), pp. 193–230. https://doi.org/10.3920/978-90-8686-803-2_9.

4. J. Csapó, T. G. Martin, Z. S. Csapo-Kiss, and Z. Hazas (1996). Protein, fats, vitamin and mineral concentrations in porcine colostrum and milk from parturition to 60 days. Int. Dairy J. *6*, 881–902. https://doi.org/10.1016/0958-6946(95)00072-0.

Supplementary Table 5. Organ weight by treatment group.

		**ALAC** (n=11)	**WPI** (n=12)	**SF** (n=6)	**ALAC** vs. **WPI**	**ALAC** vs. **SF**	**WPI** vs. **SF**
Brain	g	40.18 ± 0.80	39.84 ± 1.07	43.63 ± 1.77	ns	ns	*
	%	1.46 ± 0.09	1.47 ± 0.06	0.92 ± 0.06	ns	***	***
Liver	g	83.58 ± 5.31	75.08 ± 5.09	132.77 ± 5.90	ns	***	***
	%	2.96 ± 0.11	2.71 ± 0.08	2.76 ± 0.08	*	ns	ns
Left kidney	g	12.23 ± 1.05	13.78 ± 1.89	16.37 ± 0.88	ns	ns	ns
	%	0.43 ± 0.02	0.49 ± 0.05	0.34 ± 0.02	ns	ns	*
Right kidney	g	11.88 ± 0.99	13.12 ± 1.57	15.92 ± 0.91	ns	*	ns
	%	0.42 ± 0.02	0.47 ± 0.04	0.33 ± 0.02	ns	ns	**

Organ weight data are expressed in grams or as a percentage of body weight, expressed as mean ± SEM. Significant differences were evaluated using ANOVA following by Tukey HSD post-hoc tests, controlling for litter effects. *** $p<0.001$, ** $p<0.01$, * $p<0.1$, ns, not significant.

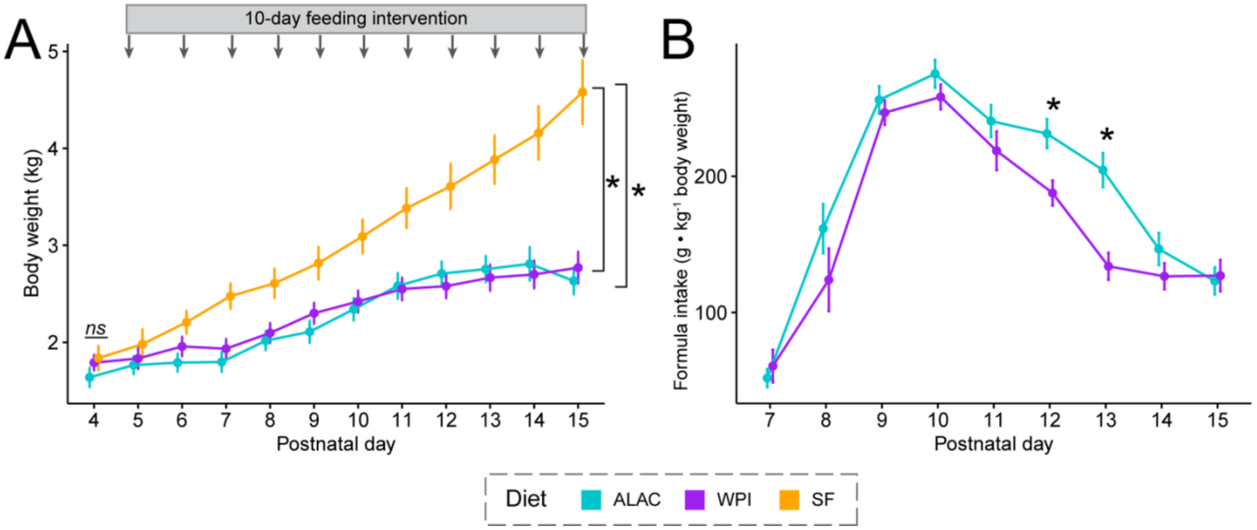

Supplementary Fig 1. Growth and formula intake.

A. Body weight from postnatal day 4 to 15. The group difference was evaluated either cross-sectionally at enrollment (postnatal day 4) or overall (from postnatal day 5 to 15) using ANOVA followed by post-hoc Tukey HSD test, accounting for the litter effect in the model.

B. Daily formula intake from postnatal day 7 to 15. The group difference was evaluated cross-sectionally using ANOVA, accounting for litter effect in the model.

Data are represented as mean ± SEM.

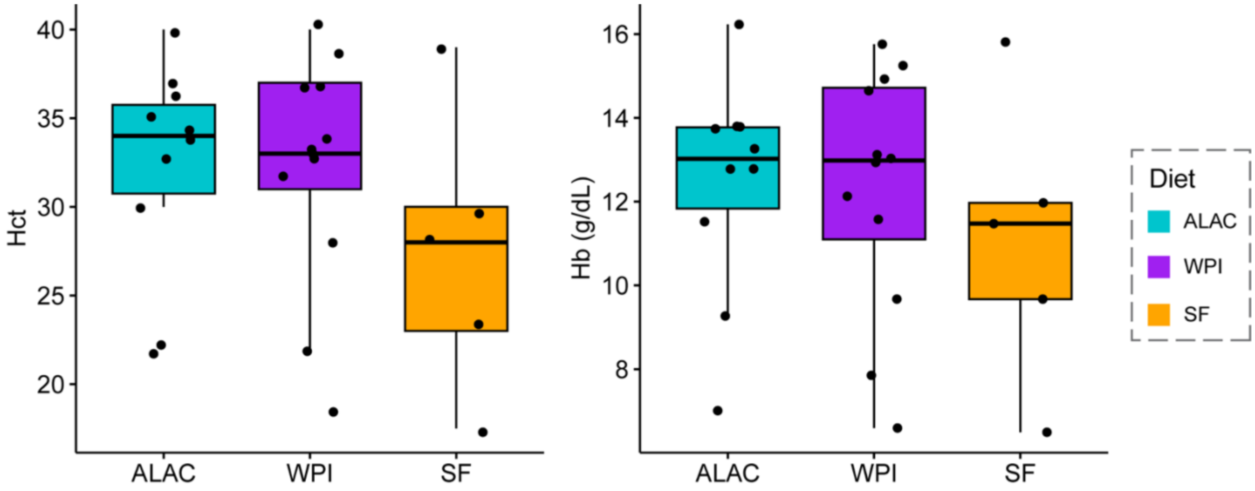

Supplementary Fig 2. Assessment of hematocrit (Hct) and hemoglobin (Hb) on Day 16.
The group difference was evaluated using ANOVA following by Tukey HSD post-hoc tests, accounting for litter effect in the model. No significant difference was found between groups. Data are represented as mean ± SEM.

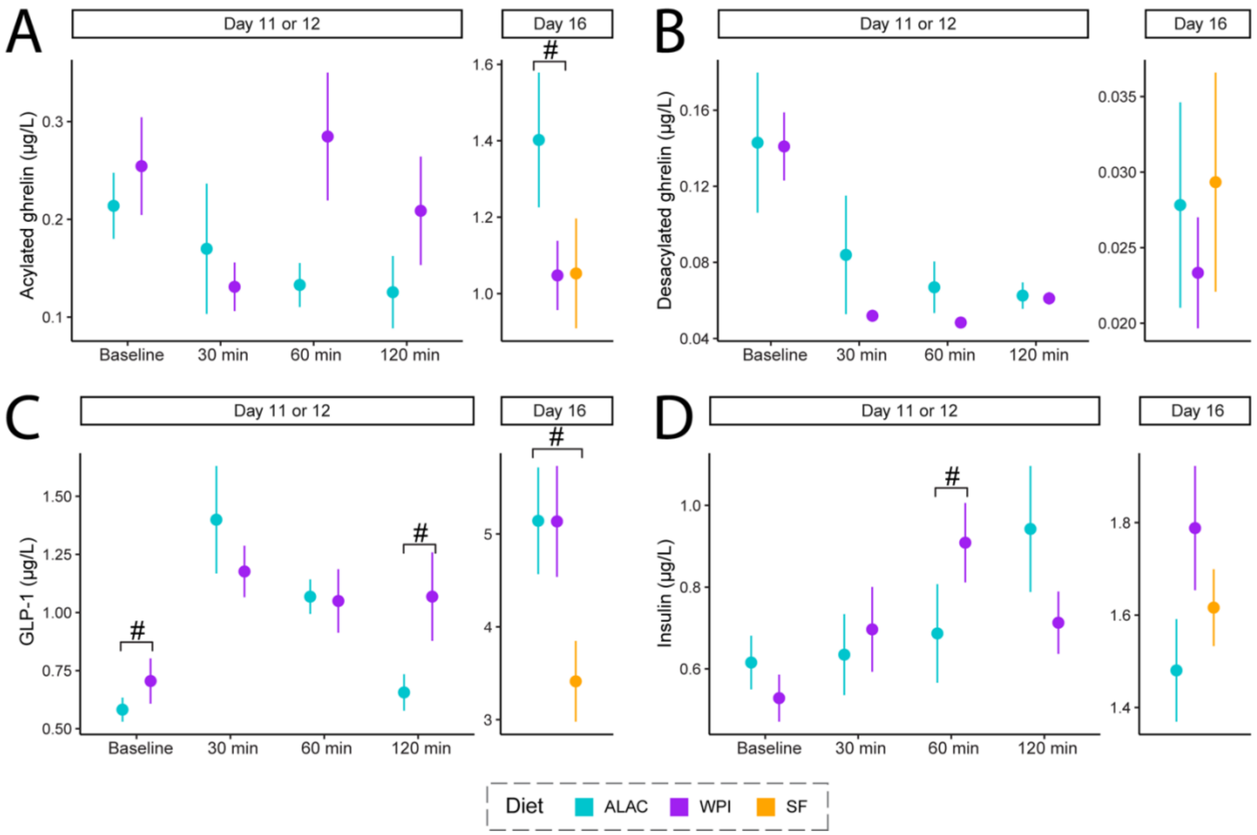

Supplementary Fig 3. The impact of an α-lactalbumin enriched formula on circulating hormones.
Acylated ghrelin, desacylated ghrelin, GLP-1 and insulin levels were assessed at baseline and at 30, 60 and 120 minutes post-meal on Day 11 or 12, as well as on Day 16. The group differences were evaluated cross-sectionally using the Mann-Whitney U test. For the ALAC group, the number of samples analyzed at each timepoint during the postprandial study (Day 11 or 12) was: baseline (n = 10), 30 min (n = 8), 60 min (n = 10), and 120 min (n = 6). For the WPI group: baseline (n = 10), 30 min (n = 7), 60 min (n = 10), and 120 min (n = 9). The number of samples analyzed at necropsy (Day 16) was: ALAC group (n=11), WPI group (n=12), and SF group (n=6). Data are represented as mean ± SEM. * p<0.05, # p<0.1.

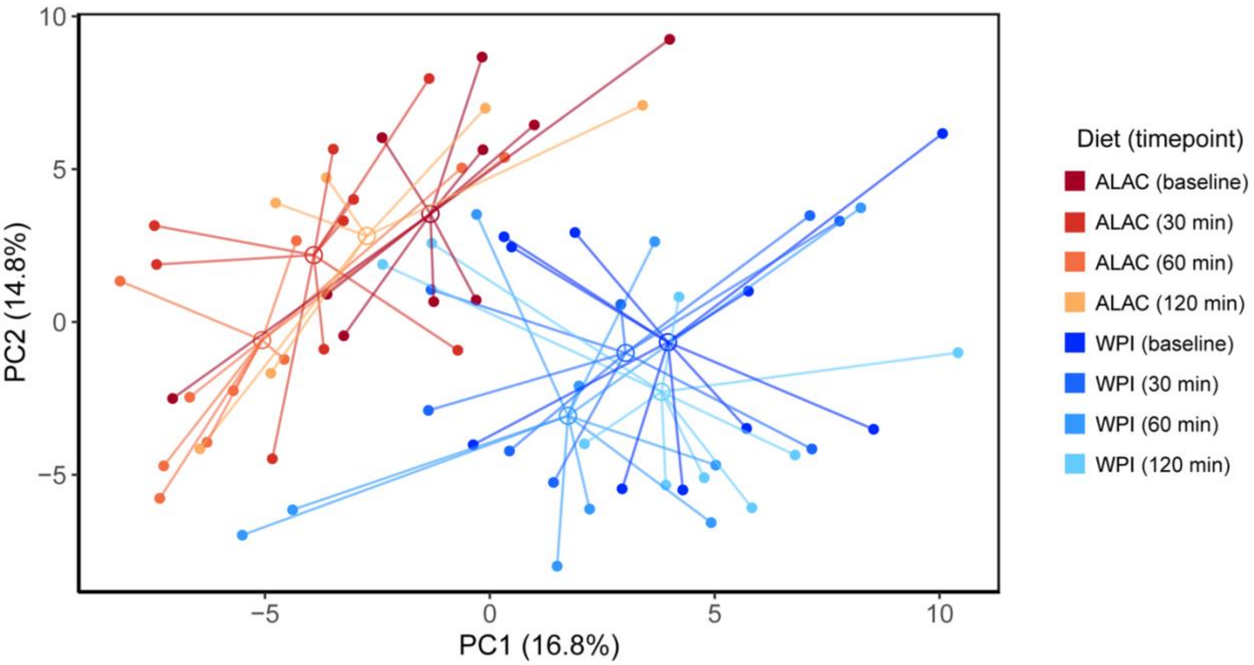

Supplementary Fig 4. The impact of α-lactalbumin on the overall postprandial serum metabolome.
Principal components analysis of the GC-MS-based untargeted serum metabolomes at baseline and at 30, 60 and 120 minutes post-meal.

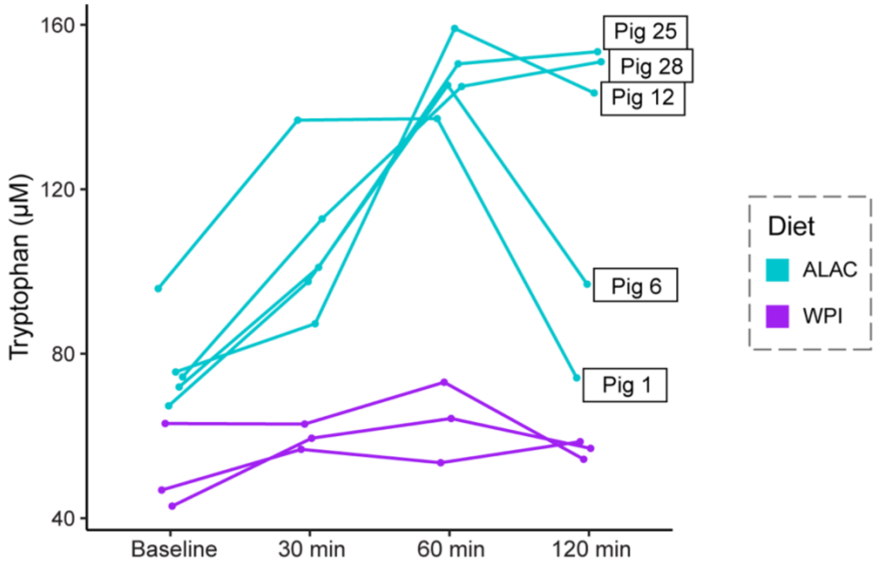

Supplementary Fig 5. Postprandial levels of tryptophan following consumption of formulas predominantly containing α-lactalbumin or β-lactoglobulin as major whey proteins.

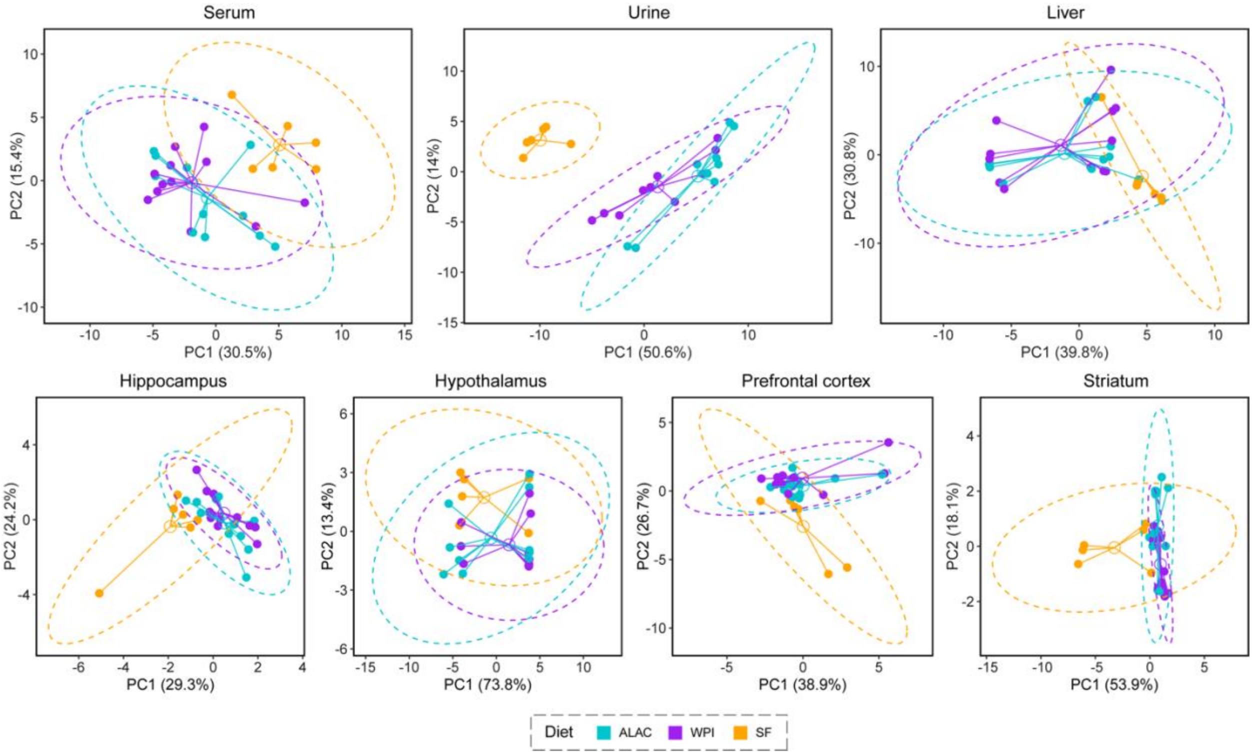

Supplementary Fig 6. Principal component analysis of serum, urine, liver and brain metabolomes on Day 16. Targeted metabolomics data was used for illustrating the results. The ellipses were constructed based on multivariate normal distribution at a 95% confidence level.

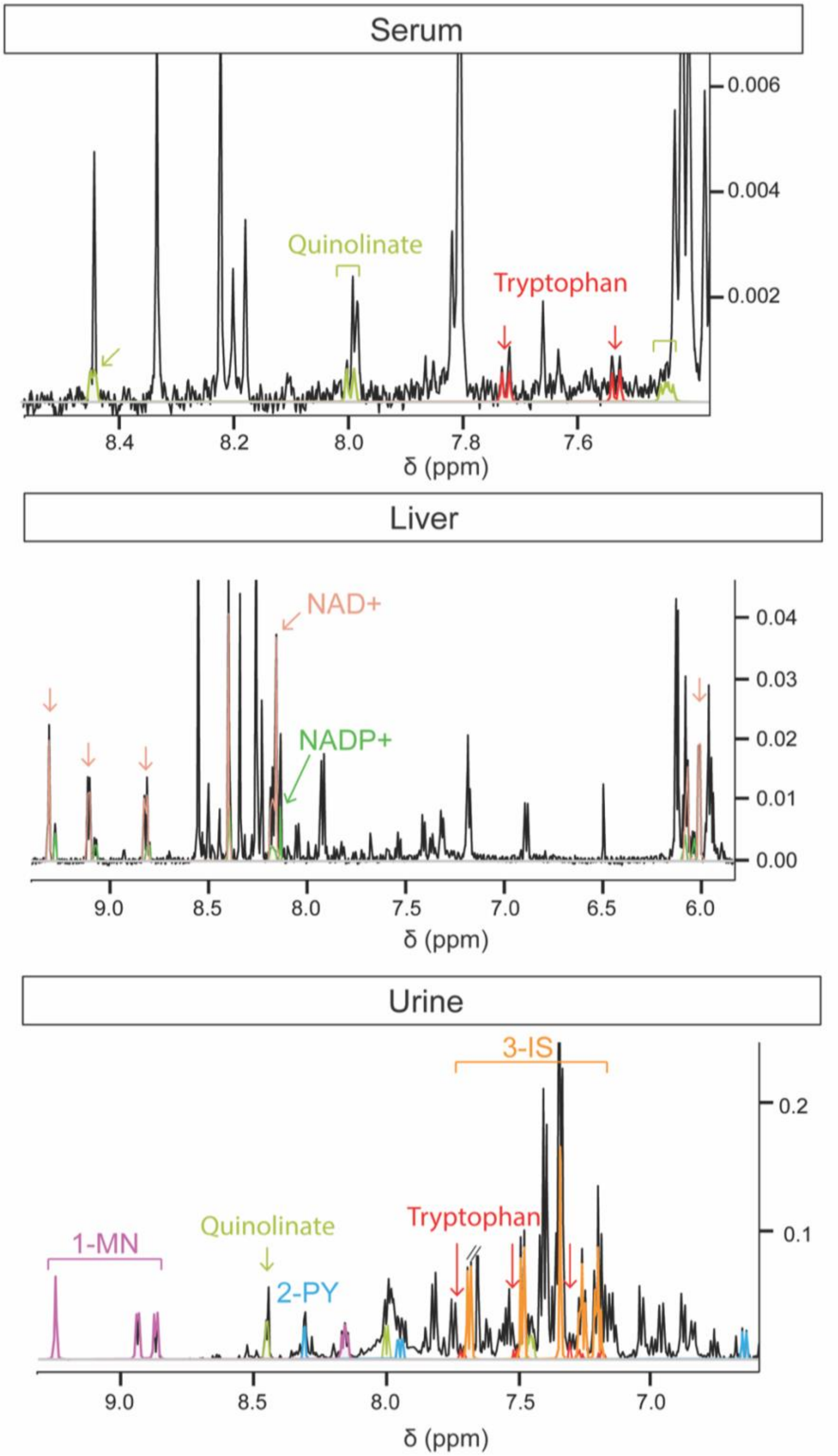

Supplementary Fig 7. Representative 600 MHz ^{1}H-NMR spectra from serum, urine and liver samples showing peak assignments for tryptophan metabolism-related metabolites quantified in this study. Quantification was performed using the Chenomx NMR Suite reference library (version 8.6). **Abbreviations**: 1-MN, 1-methylnicotinamide; 2-PY, *N*-methyl-2-pyridone-5-carboxamide; 3-IS, 3-indoxyl sulfate; NAD$^+$, nicotinamide adenine dinucleotide; NADP$^+$, nicotinamide adenine dinucleotide phosphate.
